# Surface Engineering of Ceramic Nanomaterials for Separation of Oil/Water Mixtures

**DOI:** 10.3389/fchem.2020.00578

**Published:** 2020-11-19

**Authors:** Usama Zulfiqar, Andrew G. Thomas, Allan Matthews, David J. Lewis

**Affiliations:** ^1^Department of Materials, University of Manchester, Manchester, United Kingdom; ^2^International Centre for Advanced Materials (ICAM), University of Manchester, Manchester, United Kingdom

**Keywords:** oil/water mixtures, separation, nanomaterial, superwetting, ceramic

## Abstract

Oil/water mixtures are a potentially major source of environmental pollution if efficient separation technology is not employed during processing. A large volume of oil/water mixtures is produced via many manufacturing operations in food, petrochemical, mining, and metal industries and can be exposed to water sources on a regular basis. To date, several techniques are used in practice to deal with industrial oil/water mixtures and oil spills such as *in situ* burning of oil, bioremediation, and solidifiers, which change the physical shape of oil as a result of chemical interaction. Physical separation of oil/water mixtures is in industrial practice; however, the existing technologies to do so often require either dissipation of large amounts of energy (such as in cyclones and hydrocyclones) or large residence times or inventories of fluids (such as in decanters). Recently, materials with selective wettability have gained attention for application in separation of oil/water mixtures and surfactant stabilized emulsions. For example, a superhydrophobic material is selectively wettable toward oil while having a poor affinity for the aqueous phase; therefore, a superhydrophobic porous material can easily adsorb the oil while completely rejecting the water from an oil/water mixture, thus physically separating the two components. The ease of separation, low cost, and low-energy requirements are some of the other advantages offered by these materials over existing practices of oil/water separation. The present review aims to focus on the surface engineering aspects to achieve selectively wettability in materials and its their relationship with the separation of oil/water mixtures with particular focus on emulsions, on factors contributing to their stability, and on how wettability can be helpful in their separation. Finally, the challenges in application of superwettable materials will be highlighted, and potential solutions to improve the application of these materials will be put forward.

## Introduction

There has been a growing interest in utilizing smart bioinspired materials with selective functional wettability for separation of oil/water mixtures and emulsions. Surfaces can be engineered to have an affinity to one class of fluid while being repellent to others. This implies that if an absorbent is repellent to one phase, it can effectively collect the second phase from a biphasic mixture. The inspiration of using bioinspired materials for oil/water separation is based on the so-called lotus leaf effect described by Barthlott and Neinhuis who observed that the self-cleaning property of the leaves of the lotus plant or water lily (*Nelumbo nucifera*) is due to a rough surface with microscale villi and is also waxy and therefore hydrophobic (Barthlott and Neinhuis, 1997). This hierarchical structure imparts a low surface energy to the leaf, making it extremely repellent to water and is said to be “superhydrophobic” [water contact angle (WCA) > 150°]. The evolutionary benefit to the plant is that it can efficiently reject water, and so the leaves do not sink. As water droplets roll off the leaf, it is also passively self-cleaning, which allows efficient photosynthesis.

**Figure 1:**
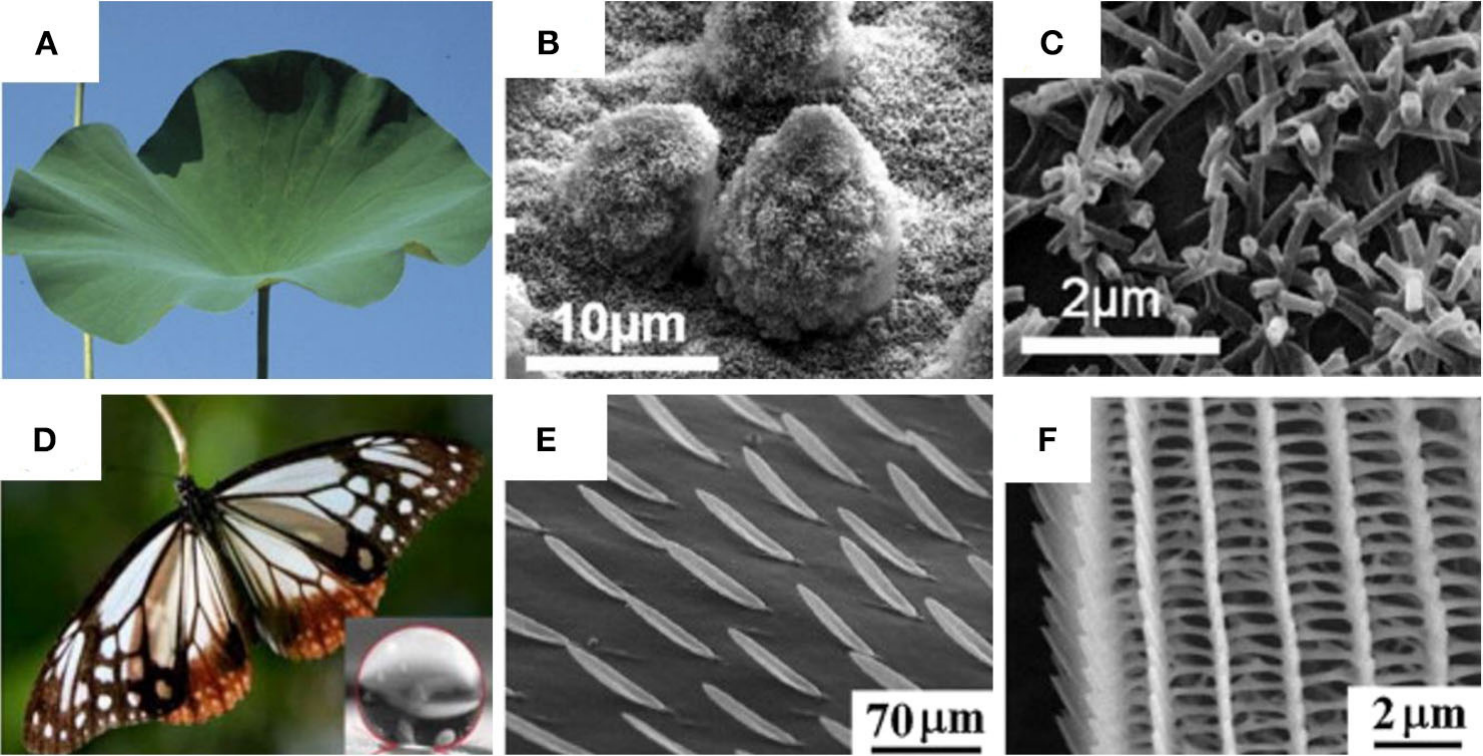
Figure showing digital images and scanning electron microscopy (SEM) images of different natural species that display hydrophobic properties: **(A–C)** *Nelumbo nucifera* (lotus); **(D–F)** *P. sita* butterfly wings. **(A–C)** Adapted from Koch et al. (2009) and **(D–F)** Adapted from Perez Goodwyn et al. (2009). With permission from Elsevier and NPG (2009), respectively.

This, of course, is not a feature exclusive to the lotus leaf; other insects and plants also have features that may display superhdyrophobic properties with varying microscale and nanoscale surface features. For example, Indian canna plants contain wax platelets, which are randomly distributed on microscale rods (WCA ~ 165°), whereas the taro leaves are composed of a surface containing nanoscale elliptic features, which are uniformly distributed on micron-sized features (WCA ~ 159°) (Crawford and Ivanova, 2015). Insects such as water striders (*Gerridae*), dragonflies (*Anisoptera*), cicadae (*Cicadoidea*), and butterflies (*Papilionoidea*) also possess anti-wetting and anti-fogging adaptations (Figure 1). The wings of the dragonfly *Hemianax papuensis* contains nanopillars, which form a fractal structure resulting in an extremely water-repellent surface (WCA ~ 161°) (Ivanova et al., 2013; Crawford and Ivanova, 2015). Since the advent of electron microscopies, which allowed the discovery of the surface features that allow the superhydrophobic phenomenon in natural species, scientists have sought to engineer artificial superhydrophobic surfaces (Ge et al., 2020b; Liao et al., 2020; Shao et al., 2020). Mimicking of superhydrophobic surfaces has since been successfully achieved in labs worldwide, and this branch of *biomimicry* has found many applications such as self-cleaning materials, protection of building materials, corrosion resistance, anti-icing, drag reduction, biomedical, and separation of oil/water mixtures and emulsions (Cheng et al., 2017; Cho et al., 2017; Gao et al., 2017b; Siddiqui et al., 2017; Tang et al., 2017a; Zhang et al., 2017b, 2018b; Ren et al., 2018; Kang et al., 2020; Lu et al., 2020a,b; Nine et al., 2020; Zhu et al., 2020). Although separation of oil/water mixtures had been demonstrated successfully using superhydrophobic surfaces, the separation of emulsions is not so easy, as the oil droplets are uniformly distributed and often stabilized within the water phase. Therefore, a range of methods involving superwettable materials have also been explored to realize separations of emulsions and in some cases surfactant stabilized emulsions (Li et al., 2016; Liu et al., 2016, 2017b; Chen et al., 2017; Wang et al., 2017a). This review covers different types of superwetting interfaces for separation of biphasic mixtures.

### Immiscible Fluidic Mixtures

#### Oil/Water Mixtures

Oil/water mixtures are produced in the petrochemical, leather, food, metallurgical, and beverage industries on a regular basis. In the oil industry, the volume of an oil/water mixture is greatly increased by enhanced oil recovery techniques during the drilling from reservoir. At the end of reservoir life, enhanced oil recovery techniques are employed to acquire 20–40% more oil. Recovering the oil from an oil/water mixture is of paramount interest to increase the economic value of operations in petrochemical industries (Yu et al., 2016). These techniques are accompanied with many drawbacks including oil/water mixtures being produced in the form of stable emulsions. Usually, oil/water mixtures do not exist as two separate phases; instead, they are produced in the form of emulsions, which are further modified with some natural surface active agents to stabilize the oil phase in water, making the separation process extremely difficult.

Organic contaminants are produced in these processes, and if released into marine and river waters, they can affect biological organisms in many adverse ways, as well as cause atmospheric pollution, destroying crops for consumption and causing a potential fire hazard (Poulopoulos et al., 2005; Yu et al., 2017a). Oil/water mixtures are a potential problem owing to the unprecedented harmful effects of hydrocarbons in water streams on the environment, on natural habitats, and on human health (Huang et al., 2015; Wan et al., 2017; Gebreslase, 2018; Shin et al., 2018).

Accidents during production or transportation of oil are another cause of oil/water mixture discharge to the environment. Major accidents such as the Exxon Valdez spill (1987), the Deepwater Horizon fire and spill (2010), and the Chennai oil spill (2017) have all been deleterious to the marine environment (Brody et al., 2012; Han et al., 2018). As the oil comes to the surface of water owing to its low density, it affects the animals that have to go through air/water interface to breathe. The rise and fall of tides also enable the contact between oil and natural species and affect them in several ways including digestion of oil, inhalation of vapors, and DNA damage (Major and Wang, 2012; Chang et al., 2014). Accidents that happen close to the shore not only are difficult to clean but also have adverse effects on human health and economy.

#### Emulsions in the Petrochemical Industry

An emulsion is a metastable mixture of two immiscible fluids where droplets of one liquid are distributed in the another liquid phase (Wong et al., 2015). These types of mixtures are mostly formed in the presence of a third phase termed as emulsifier, which could be either a surfactant molecule or solid particles. Emulsions have been studied extensively in the past few decades, and several definitions and classification of these systems exist. For example, they have been defined as a mixture of two immiscible liquids with one phase uniformly distributed in the other with the help of an emulsifying agent (Aziz et al., 2002; Umar et al., 2018). They are thermodynamically unstable mixtures, which will eventually separate from each other in order to reduce the interfacial area. There exist different classifications of emulsions such as oil in water, water in oil, and complex emulsions. Oil-in-water emulsions are formed when the oil droplets are dispersed in water, whereas water-in-oil emulsions are formed when water droplets are dispersed in oil. In complex emulsions, water is dispersed in oil, which is also mixed in a continuous water phase (water/oil/water emulsion). Emulsions are stabilized by the presence of emulsifying agent, and the balance of lipophilic and hydrophilic properties of the agent is one of the important factors in dictating the emulsion type. The emulsifying agents are the surface active agents, which make the interfacial film leading to the reduction of interfacial tension and suspension of the droplets. Emulsions are also characterized as tight (small droplets) or loose (large droplets) on the basis of the size of droplets dispersed in a continuous phase. According to Becker, an emulsion can be called a *macroemulsion* if the size of droplets is bigger than 0.1 μm and a *microemulsion* if the size of dispersed droplets is <0.1 μm (Becker, 1997).

Emulsion generation is a serious problem in several industrial operations such as pharmaceuticals, cosmetics, food, and especially the petrochemical industry, where emulsions are formed at the well bore, during enhanced oil recovery operations and during downstream processing (Ghosh and Rousseau, 2010). These emulsions often contain salts, which may cause corrosion in pipelines. The amount of water is increased in crude oil near the end of the life of a reservoir (Lim et al., 2015), and both immiscible liquids undergo intense pressure, causing mixing and the generation of emulsions (Sjöblom et al., 2003). Emulsification like this makes cleanup difficult in the event of an oil spill, as it alters the viscosity and density of oil. It can also enhance the volume of a mixture as observed in previous studies, where an increase in volume of spilled material up to five times has been observed owing to emulsification (Fingas and Fieldhouse, 2003, 2004; Wong et al., 2015). Crude oil also contains several minerals and compounds such as asphaltene, organic acids, solid particles, organometallics, corrosion products, and waxes; and some of these compounds help to create a stable emulsion with water. Solid particles of clays, silica, and iron oxide are naturally hydrophilic but can become hydrophobic after long contact with oil. This, combined with the presence of asphaltenes and resins, can help water droplets to uniformly disperse in oil or vice versa, generating very stable emulsions.

## Separation of Oil/Water Mixtures

There are several approaches to separate oil/water mixtures in different situations. The US Environmental Protection Agency classifies the separation in three different categories in the event of an oil spill. These include mechanical recovery (booms, skimmers, and sorbents), chemical and biological methods (gelling agents, dispersants, and biological agents) and physical separation (pressure washing and sorbents) (US EPA). *In situ* burning of oil is sometimes used for urgent treatment of oil spills but is limited by many factors (Ornitz and Champ, 2002). For example, if the oil contains a significant amount of water, then ignition becomes very difficult; moreover, gases produced as a byproduct of combustion may cause secondary environmental pollution. There is a natural tendency in certain organisms to consume hydrocarbons for the production of carbon and energy to create new microbial cells (Ivshina et al., 2015), which could be an ecologically viable and elegant way to remove oil from oil/water mixtures; however, these processes are slow and rely on conditions conducive to the microbes used.

Floatation and coagulation are some of the more widely used methods to treat emulsions. In floatation, air bubbles are introduced, which become attached to the light fractions of the mixture (oil or particles) and transport them to the surface. Flotation is more beneficial as compared with gravity separation in terms of efficiency for removal of small particles (Saththasivam et al., 2016). Starting from the generation of air bubbles of specific size, which become attached the oil droplets, this is followed by aggregation of droplets, which subsequently float to the top surface to form a layer that can be skimmed (Aulenbach et al., 2010). Coagulation is another method in which chemical agents are added to moderate the surface charge of oil droplets to help them aggregate and settle. The oil droplets are negatively charged owing to adsorption of moieties, which restrict their aggregation. Some chemical agents with opposite charge are introduced in the system, which mitigates the charges on oil droplets and destabilize the colloids, promoting their coalescence. For example, long-chain polymers have been introduced, which act as a bridge to connect different colloidal particles to assist in the aggregation (Jiang, 2015; Yu et al., 2017a; Sillanpää et al., 2018; Wei et al., 2018).

Other approaches toward separation are the use of mechanical devices such as booms, skimmers, or centrifugal devices such as hydrocyclones. Physical separation of the immiscible mixtures using absorbents is advantageous from many perspectives; however, traditional absorbents face limitations owing to their affinity toward both oil and water. The absorbent may not be efficient in the separation of emulsions, especially those stabilized with surfactants. A method to improve the efficiency of an absorbent is to engineer the surface properties to tune their wettability toward a particular liquid type. For example, if an absorbent is to be made oleophilic and hydrophobic, it will only absorb oil from an oil/water mixture, leaving behind the clean water. Generally, the adsorption starts by the interaction of oil droplets with a sorbent surface followed by its entrapment in the porous framework of the sorbent. This is one of the easiest methods to separate the oil/water mixtures; however, the surface properties of adsorbents greatly influence their interaction with liquid.

Physical separation using membranes is a simple approach to physically separate the mixtures without using additional chemicals. The membrane acts as a porous layer and controls the transportation between oil and water (Padaki et al., 2015), and can also block the penetration of solids through their pores whilst letting the liquid pass through. Sorbents and membranes are useful materials for the remediation of oil/water mixtures and physical collection of oil. The surface engineering of these materials to tune their affinities toward oil or water is extremely important for efficient separation. The wetting properties of a surface such as high repellency toward water (superhydrophobic), an affinity toward water (superhydrophilic), high repellency toward oil (superoleophobic), and an affinity toward oil (superoleophilic) mainly depend upon the chemical structure and geometry of the surface. Surface-engineered absorbents and membranes can be an effective tool for physical separation of oil/water mixtures and emulsions.

### Surface Properties for Physical Separation

The surface of a material can be engineered to have a combination of wetting properties so as to be used for separation of oil/water mixtures. This combination could be superhydrophobic/superoleophilic (Han et al., 2017; Sun et al., 2018) or superoleophobic/superhydrophilic (Li et al., 2018a; Qu et al., 2018), depending upon the type of material and nature of application environment. Generally, surfaces with superhydrophobic properties are used to repel the water in a mixture and attract the oil, whereas the opposite arrangement is used to repel oil from oil/water mixtures, which utilize superoleophilic surfaces. Hence, if an absorbent collects oil from an emulsion while repelling water, it will have a surface that is both superhydrophobic and superoleophilic. For example, hierarchically structured MnO_2_ particles have been used to separate oil from emulsions (Guo et al., 2017a). The particles were modified with stearic acid to render them superhydrophobic and superoleophilic.

A detailed theoretical background of surface wettability is provided in the Supplementary Information.

### Superhydrophobic/Superoleophilic Surfaces

Generally, the materials with these properties have been constructed by coating porous substrates with a superhydrophobic coating. A superhydrophobic coating can provide excellent resistance to water but allows the permeation of low surface tension liquids. When water is introduced to the surface of these materials, it forms a spherical droplet (CA > 150°) while an oil droplet completely spreads over it (CA < 10°). These types of surface are mostly inspired by nature, as several natural species have the ability to repel water owing certain physicochemical features of their surface (e.g., lotus leaf, *vide supra*). Superhydrophobic surfaces can be created by combining nanoscale hierarchical roughness with low-surface-energy materials. For example, fluorosilane-modified silica nanoparticles have been used in combination with epoxy resin to create an artificial superhydrophobic surface. The nanoscale particles helped to achieve a hierarchical structure with a low surface energy (Jia et al., 2019). Recently, a dual-scale modification was carried out on a melamine sponge to create a combination of superhydrophobic and superoleophilic properties (Lv et al., 2019). The sponge was coated with graphene oxide (GO) followed by decoration with hydrophobic magnetite nanoparticles. After the modification, the sponge was able to effectively collect oil while rejecting the water. There are other examples where this combination of properties has been used to engineer materials for oil/water separation (Zhu et al., 2017; Ge et al., 2019, 2020a). A variety of nanomaterials have been used to create meshes (Jiang et al., 2017; Zhang et al., 2018a, 2019; Nanda et al., 2019; Wang et al., 2019; Gong et al., 2020), membranes (Attia et al., 2018; Huang et al., 2018; Ma et al., 2018; Qing et al., 2018; Zhao et al., 2018; Subramanian et al., 2019; Cheng et al., 2020), sponges (Huang et al., 2019b; Li et al., 2019b), and fabrics (Cheng et al., 2018; Chauhan et al., 2019; Lin et al., 2019; Zhou et al., 2019; Dan et al., 2020) with superhydrophobic and superoleophilic properties for oil/water separation (Ferrero et al., 2019; Zulfiqar et al., 2019; Latthe et al., 2020; Lin et al., 2020; Topuz et al., 2020; Zhang et al., 2020b). Most of these materials separate oil either by filtration, absorption, or both. These materials have certain limitations, as most of the separation is gravity driven in the case of filtration, which could be very slow or may not even work in the case of emulsions. Another problem is fouling of membranes or mesh pores by oil adsorption. The solid content in oil especially in the case of crude oil can easily block the pores affecting the whole separation process. In the case of oil-in-water emulsions, when they are stabilized by a surfactant, the oil is uniformly distributed in the water phase and does not come in direct contact with separation materials, thus making it ineffective for separation. However, there is recent trend in engineering microscale absorbents with superhydrophobic and superoleophilic properties, which can go into the emulsion under mechanical agitation and collect the dispersed oil phase from it (Duan et al., 2015; Guo et al., 2017a,b; Chen et al., 2018). This approach is reported to be effective for removal of oil in several scenarios (present on the surface of water or uniformly distributed in water). Further details on surface engineering of different materials for this approach will be discussed in the next sections.

### Superoleophobic/Superhydrophilic Surfaces

Rendering superoleophobic properties to a surface is not simple, because there exists a vast difference in surface tensions of water and organic liquids. For example, water has surface tension of 72.8 mN m^−1^, whereas the surface tension of *n*-hexane is 18.4 mN m^−1^; therefore, recently, some strategies such as modification of different nanomaterials with fluorosilane (Kwak and Hwang, 2016; Dong and Zhang, 2018) engineering of re-entrant surface geometry for superoleophobic properties (Nosonovsky and Bhushan, 2016) and underwater superoleophobicity have been studied in detail (Hong et al., 2018; Obaid et al., 2018). From an application's point of view, superoleophobic surfaces can be divided into several categories; however, this review is focused on surfaces that have both superoleophobic and superhydrophilic properties. This combination of properties can be used for oil/water separation via rejection of oil while allowing water to pass through. In a typical example, a surface with a combination of superoleophobic and superhydrophilic properties was formed by using titania nanoparticles and fluorinated compounds containing hydrophilic units (Na^+^); and its application in oil/water separation was demonstrated (Li et al., 2018a). Titania nanoparticles and fluorinated compounds containing hydrophilic units (Na^+^) were used to construct these coatings on substrates such as steel mesh, sponge, and cotton. The hydrophilic unit bestows hydrophilic properties, whereas fluorine groups are responsible for the oleophobic character of the coating. The coatings were used to separate stabilized emulsion and mixtures of water and vegetable oil. Similarly, nanoparticles with thiol–acrylate components were used to create a coating with similar properties (Xiong et al., 2018).

Introducing liquid into a nanostructrure is a different approach to achieve the combination of superoleophobic and superhydrophilic properties. This method is inspired by fish scales, which possess oil-repellent properties in water, enabling them to swim in oil-contaminated water. The fish scales are made of protein and calcium phosphate, which is a hydrophilic material. These scales have an affinity toward both water and oil in air; however, once water is penetrated in this structure, it becomes superoleophobic with an oil contact angle of 156° (1,2-dichloroethane). There are micropapillae with rough structure, which are distributed on the surface of fish skin, which, when soaked with water, provides a superoleophobic platform. Several types of artificial materials, especially membranes, have been synthesized with underwater superoleophobic properties for oil/water separation (Zhao et al., 2016; Yong et al., 2017; Qian et al., 2018; Wang et al., 2018; Wu et al., 2018). Recently, Ni(OH)_2_ particles have been coated on a fabric to create a hierarchical porous platform (Wang and Wang, 2019). The composite fabric is superhydrophilic while demonstrating superoleophobic properties underwater. Moreover, the fabric can be made superhydrophobic after modification with stearic acid. The fabric was able to separate oil/water mixtures with a separation efficiency of more than 96% for both light and heavy oils. In another approach, Cu(OH)_2_ was deposited on steel felts, which had an underwater contact angle of 154° for *n*-octane. The material was able to separate the oil/water mixtures by allowing the water to permeate while retaining oil.

### Progress in Surface Engineering of Ceramic Nanomaterials

#### Silica-Based Materials

In silica (SiO_2_), the silicon atom is surrounded by four oxygen atoms to form a tetrahedral vertex, which is further bridged with others by a shared oxygen atom to form a three-dimensional network. Silica possesses a unique combination of properties, which make it a material of choice for several applications. For example, it has thermal stability of up to 1,500°C along with good mechanical strength. Moreover, the silanol groups (Si–OH) present on the surface of silica make it a suitable support for the grafting of several moieties. The synthesis of silica nanoparticles is generally carried out by sol–gel processing by using silicates or alkoxides (Zulfiqar et al., 2015; Khan et al., 2016). The steps in synthesis of nanoparticles include hydrolysis of precursors followed by condensation. A typical sol–gel process is sometimes combined with other modifications to produce silica nanomaterials of desirable features. For example, the templates such as polystyrene (PS) and chitosan have been utilized during synthesis of silica by a sol–gel process to create desired shapes in final particles (Lalchhingpuii and Lalhmunsiama, 2017; Rahman et al., 2017). Hydrolysis greatly depends upon several factors including the type of precursor, pH, temperature, and additives. The ease of shape control by using different templates and reaction parameters makes silica a material of choice for several applications including drug delivery, water purification, bioimaging, and selectively wettable surfaces for oil/water separation. With regard to the oil/water separation, silica has been used in various forms such as nanoparticles, nanostructured microparticles, and nanostructure scaffolds (Anjum et al., 2020; Pirzada et al., 2020; Wang et al., 2020b).

Silane modification is therefore performed to alter the hydrophobic character of silica by replacing hydroxyl bonds with the silane functional group. In a typical modification process, OH groups present on the surface of silica react with the chlorine of chlorosilane leading to replacement of surface functional groups (Ebrahimi et al., 2017). The hydrophobic character combined with the nanoscale features creates a hierarchical structure suitable to impart superhydrophobic properties.

#### Nanoparticles

**Figure 2:**
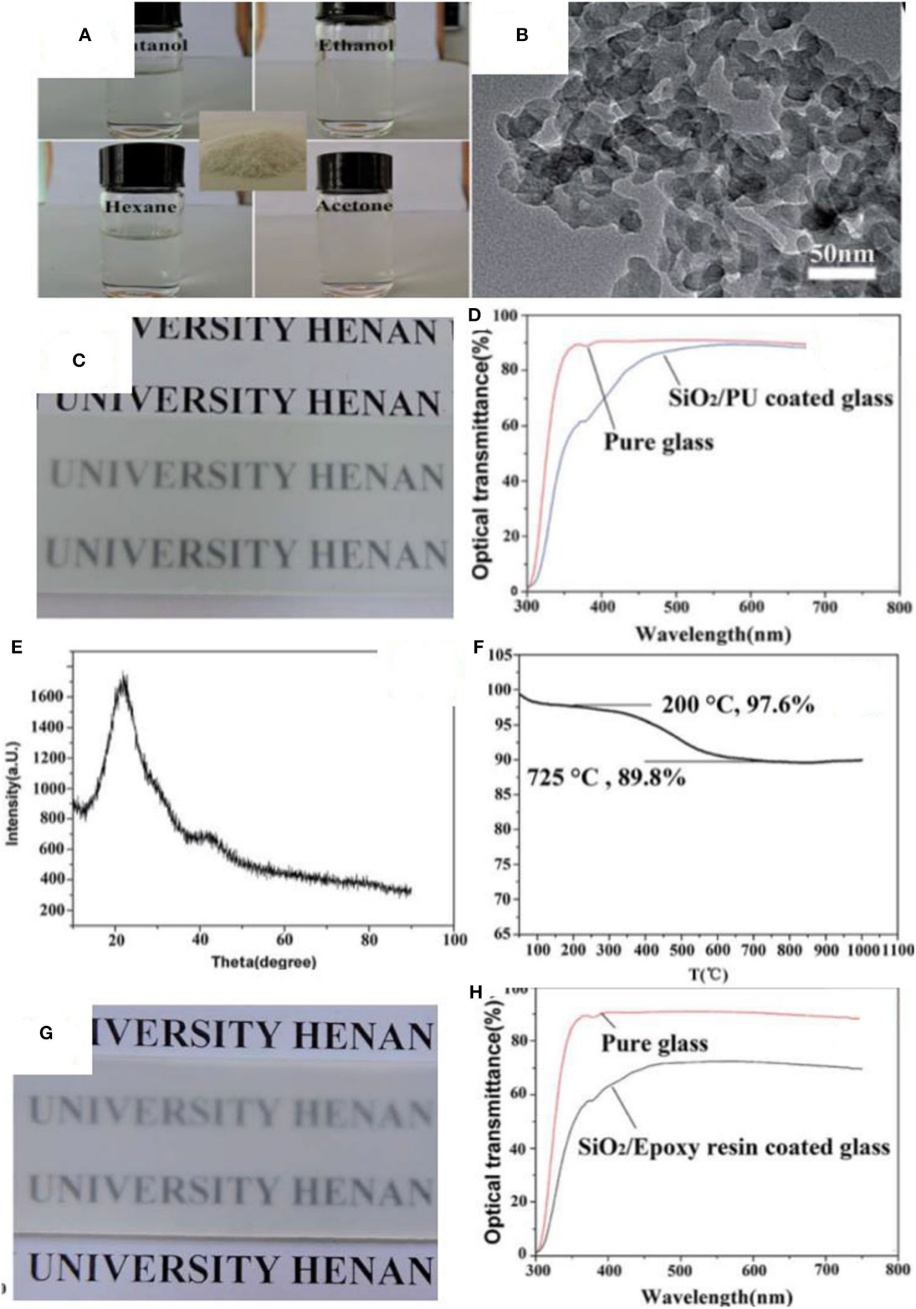
**(A)** Digital images showing silica nanoparticles dispersion in different solvents, the inset shows the silica powder. **(B)** Transmission electron microscopy (TEM) of silica nanoparticles. **(C)** Digital image showing the coating of silica/polyurethane on glass. **(D)** Difference in transmittance of glass slide before and after coating silica/polyurethane. **(E)** X-ray diffraction spectrum of silica nanoparticles. **(F)** Thermal analysis of the silica nanoparticles. **(G)** Digital images showing the coating of silica/epoxy resin on glass. **(H)** Difference in transmittance of glass slide before and after coating of silica/epoxy. Adapted from Zhi et al. (2017). With permission from the Royal Society of Chemistry, 2017.

Silica nanoparticles have been used to develop materials for the separation of oil/water mixtures (Cho et al., 2017; Kusworo et al., 2017; Ma et al., 2017; Su et al., 2017; Yang et al., 2017b; Zhang et al., 2017a; Li et al., 2018b). For example, silica nanoparticles in the size range of 40–60 nm along with the silane coupling agent hexadecyltrimethoxysilane were coated on a filter paper via a dip coating method to prepare a hydrophobic separation medium for oil/water mixtures (Tang et al., 2017b). Similarly, spherical silica nanoparticles with a diameter of 20 nm were modified with octadecyltrichlorosilane followed by deposition on the surface with the aid of polyfluorowax to make a superhydrophobic textile, which effectively separated a mixture of hexadecane and water (Zhu et al., 2017). In another study, silica nanoparticles of a diameter of 12 nm were modified at their surface with octylsilane and were decorated on a polyurethane (PU) sponge to construct a superhydrophobic platform for adsorption of oil from oil/water mixtures (Salehabadi et al., 2017). Recently, silica nanoparticles were used in combination with epoxy resin to form superhydrophobic coatings by spray deposition. Nanoparticle surfaces were modified by reaction with hexamethyldisilazane (HMDS) during synthesis (Zhi et al., 2017). It was suggested that HMDS produces trimethylsilyl and ammonia, which further reacts with hydroxyl groups of silica nanoparticles to form a layer on its surface. The introduction of the trimethylsilyl group introduces steric hindrance, which prevents the agglomeration and growth of silica nanoparticles. The hydrophobic nature of particles enabled them to be dispersed in an organic solvent as shown in Figure 2A. Transmission electron microscopy (TEM) and powder X-ray diffraction (PXRD) suggested that the silica clusters (Figure 2B) were amorphous (Figure 2E). Thermogravimetric analysis (TGA) profiles show a significant weight loss (7.8 wt.%) in the temperature range of 200–725°C (Figure 2F), which the authors ascribe to the loss of functionalities from the surface of silica. Superhydrophobic materials were formed by depositing the silica nanoparticles in combination with an adhesive (PU) onto a glass surface (Figure 2C). Figure 2G shows the coating formed by silica nanoparticles and epoxy. The difference in transmittance of these coatings is shown in Figures 2D,H; the coating formed by PU and silica showed better transmittance.

**Figure 3:**
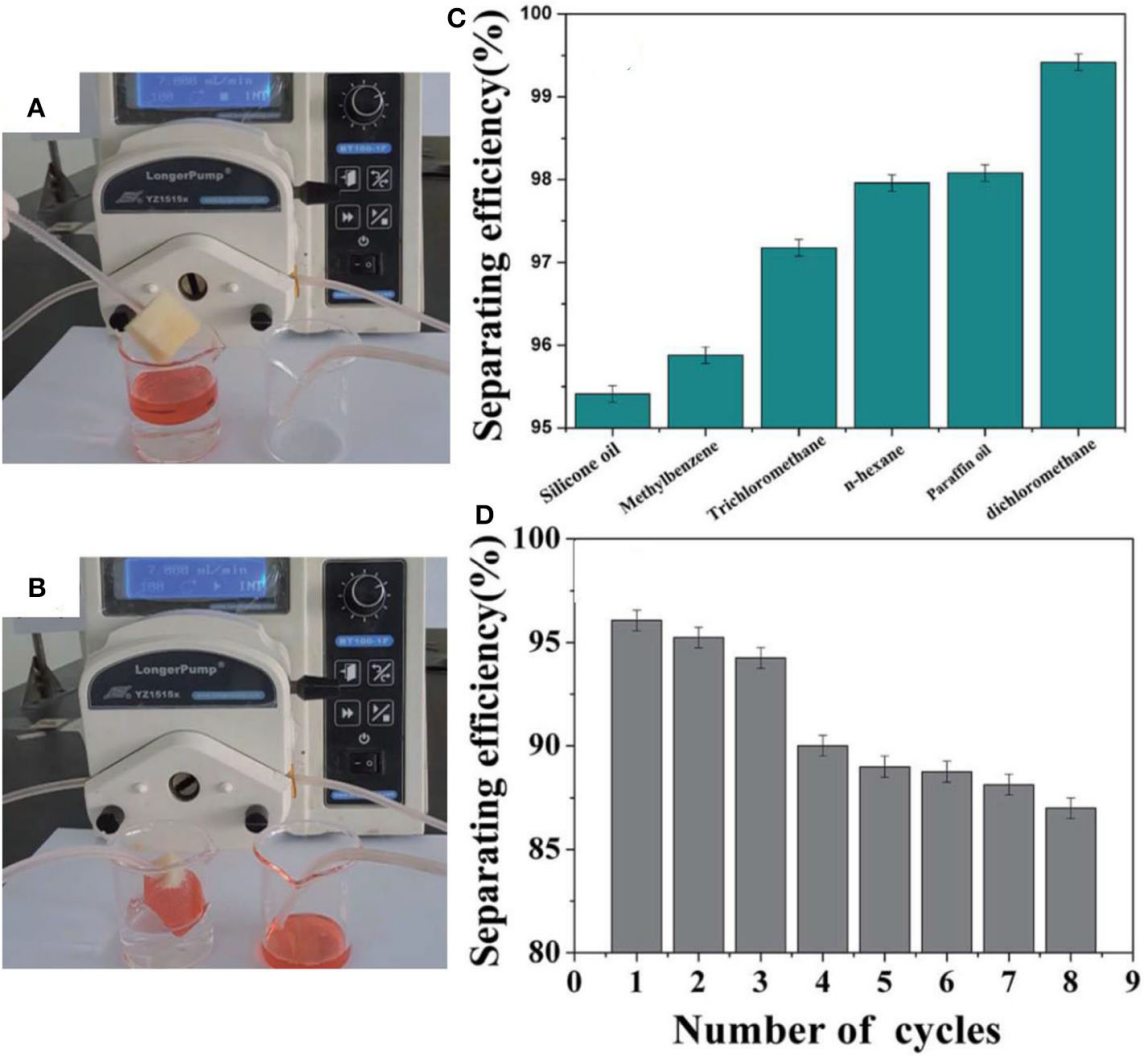
Digital images showing **(A,B)** the absorption of red-dyed silicone oil. **(C)** Separating efficiency and **(D)** recyclability of the sponge. Adapted from Zhi et al. (2017). With permission from the Royal Society of Chemistry, 2017.

The coatings were applied to several substrates including glass, fabric, paper, and sponge. The WCA in all cases was more than 150°. A superhydrophobic sponge was prepared, which was used for the separation of oil/water mixtures by mounting in a vacuum system (Figures 3A,B). The separation efficiency was observed to be more than 95% in most cases, as shown in Figures 3C,D. The separation efficiency was measured by calculating the difference in volume of liquids before and after separation.

In addition to the superhdyrophobic materials, silica nanoparticles have also been used to engineer underwater oleophobic separation media for oil/water separations. For example, a superhydrophilic and underwater superoleophobic fabric was prepared by employing a vapor–liquid interfacial reaction to decorate thiol-ene/silica hybrids on fabrics (Li et al., 2018b) (More details can be found in Supplementary Information).

#### Nanostructured Microparticles

**Figure 4:**
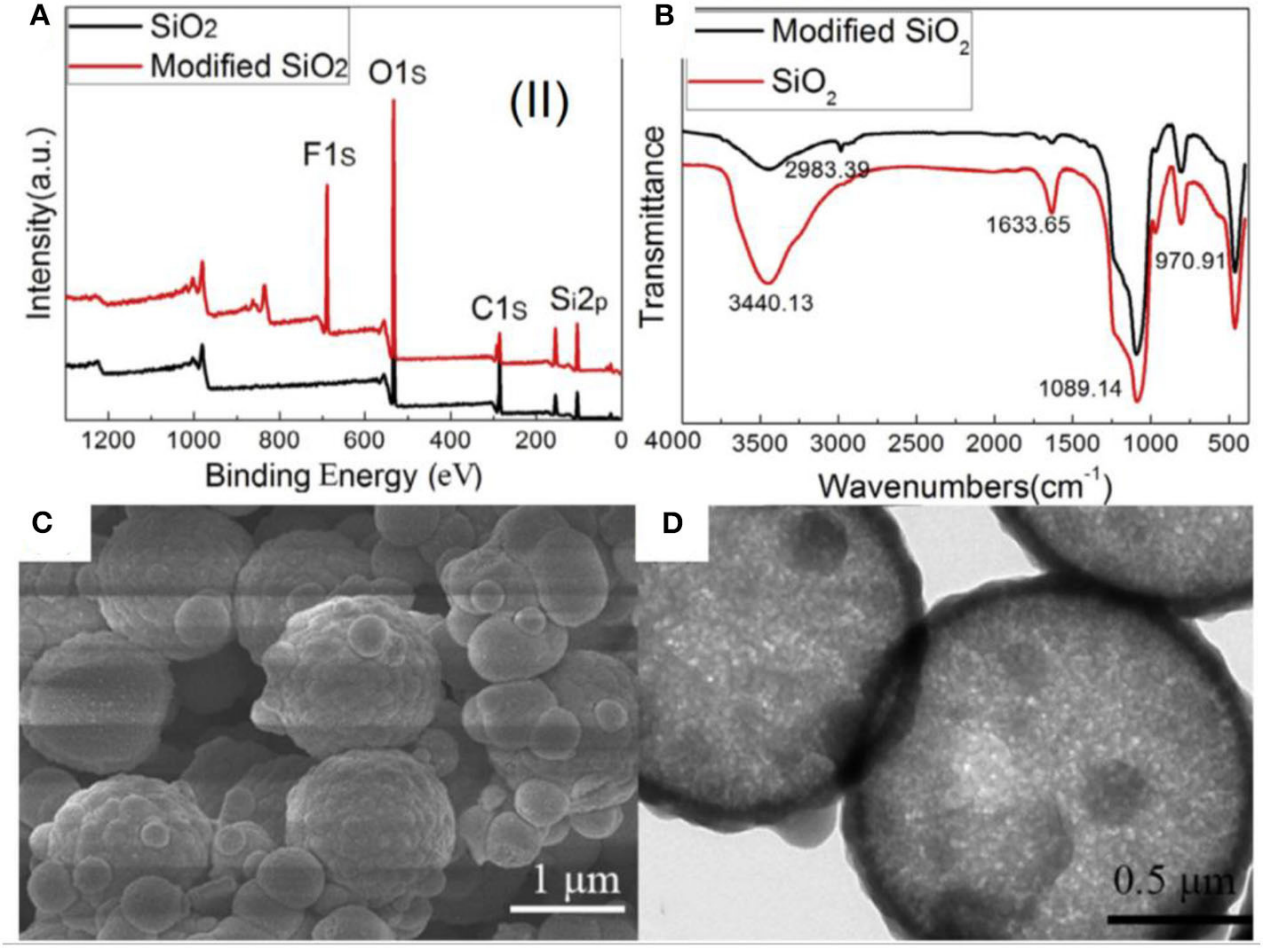
**(A)** X-ray photoelectron spectroscopy (XPS) spectra and **(B)** Fourier transform infrared (FTIR) spectra of silica before and after modification. **(C,D)** Scanning electron microscopy (SEM) and transmission electron microscopy (TEM) images of superhydrophobic silica microspheres. Adapted from Guo et al. (2017b). With permission from Elsevier, copyright 2017. Figure showing the characterizations of functionalized silica particles prepared by using polystyrene (PS) microspheres as a template.

Nanostructured microparticles in different shapes (hollow, hierarchical) have been synthesized using sol–gel chemistry and have been used for the separation of oil/water mixtures and emulsions. For example, silica particles were prepared by using polystyrene (PS) microspheres as a template (Guo et al., 2017b). Briefly, the surface of PS was made positively charged by hexadecyltrimethylammonium bromide (CTAB) so as to coat silica on it by hydrolysis of tetraethoxysilane (TEOS) in an alkaline environment. The silica particles on micro-PS particles created a hierarchical structure, which was retained even after removal of PS particles via calcination as shown in Figures 4C,D. Hollow microspheres of silica were formed, which were further modified with 1*H*,1*H*,2*H*,2*H*-perfluorodecyltriethoxysilane (PDES) to make them superhydrophobic. The PDES assembles on the surface and lowers the surface free energy, thus changing the wettability of particles.

The modification of a silica surface by self-assembly of the low-energy material was confirmed by X-ray photoelectron spectroscopy (XPS) and Fourier transform infrared (FTIR) spectroscopy (Figures 4A,B). The XPS survey spectrum indicated the presence of fluorine, which suggested the modification of pristine silica by PDES. Moreover, the intensity of hydroxyl groups in FTIR was observed to decrease after modification, which is another evidence of functionalization of silica, as pristine silica demonstrates a very strong band in this range owing to the presence of hydroxyl groups on its surface.

**Figure 5:**
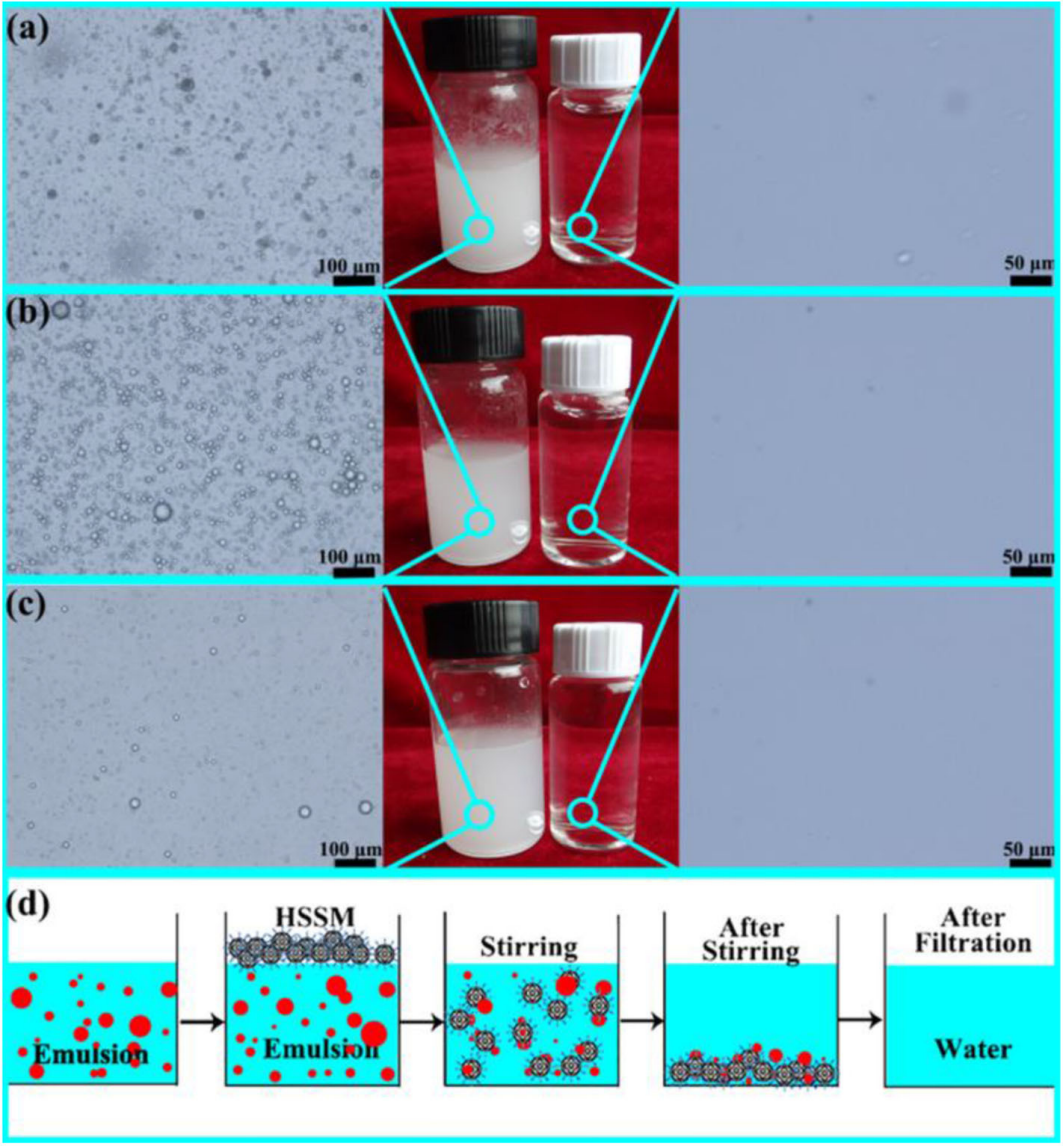
**(a)** Optical microscopy of lubricating oil-in-water emulsions stabilized by sodium dodecylbenzene sulfonate (SDS) before (left) and after separation (right). Digital images of emulsion and separation product (middle) **(b)**. Optical microscopy of diesel oil-in-water emulsions stabilized by SDS before (left) and after separation (right). Digital images of emulsion and separation product (middle) **(c)**. Optical microscopy of n-hexadecane in water emulsions stabilized by SDS before (left) and after separation (right). Digital images of emulsion and separation product (middle) **(d)**. Schematic showing the separation process of oil/water emulsions **(d)**. Adapted from Guo et al. (2017b). With permission from Elsevier, copyright 2017.

The superhdyrophobic powder was produced to fabricate superhydrophobic surfaces on different substrates. A sponge coated with the superhdyrophobic powder was used to demonstrate the separation of oil/water mixtures, whereas the modified powder was used for the separation of oil/water emulsions (Figure 5).

Oil-in-water emulsions were prepared by using diesel oil (Figure 5a), lubricating oil (Figure 5b), and hexadecane (Figure 5c). The oils were mixed with water in volume ratio of 1:100 followed by addition of 0.1 g/L of sodium dodecylbenzene sulfonate. The stable emulsions were obtained by vigorously stirring the mixture. For the separation of emulsion, the superhydrophobic powder was introduced under vigorous stirring. The microspheres captured the oil phase from the surfactant stabilized emulsion owing to the combination of superoleophilic and superhydrophobic properties. The opaque emulsion was turned clear, which indicates that the microspheres removed the oil, leaving the water behind. Optical microscopy of the emulsified solution before and after separation demonstrated that most of the oil phase was removed by the superhdyrophobic microspheres.

Similarly, PS was used in another study as template to fabricate raspberry-like silica microspheres (Yu et al., 2017b). Similar to the previous study, TEOS was used as a precursor of silica, and the surface roughness was controlled by tuning the concentration of ammonia, water, and molecular weight of polyvinylpyrrolidone. However, the PS beads were not removed and used for dual-scale roughness on the surface. The surface of silica microspheres was rendered hydrophobic by modification with hexadecyltrimethoxysilane. The modified particles were used to generate superhydrophobic surfaces on various substrates including steel mesh, paper, cotton, and sponge. The particles were anchored on a steel mesh to devise a platform for separation of oil/water mixtures. The superhydrophobic mesh separated mixtures of water and chloroform with a separation efficiency of more than 95%, which was achieved up to five cycles of separation.

**Figure 6:**
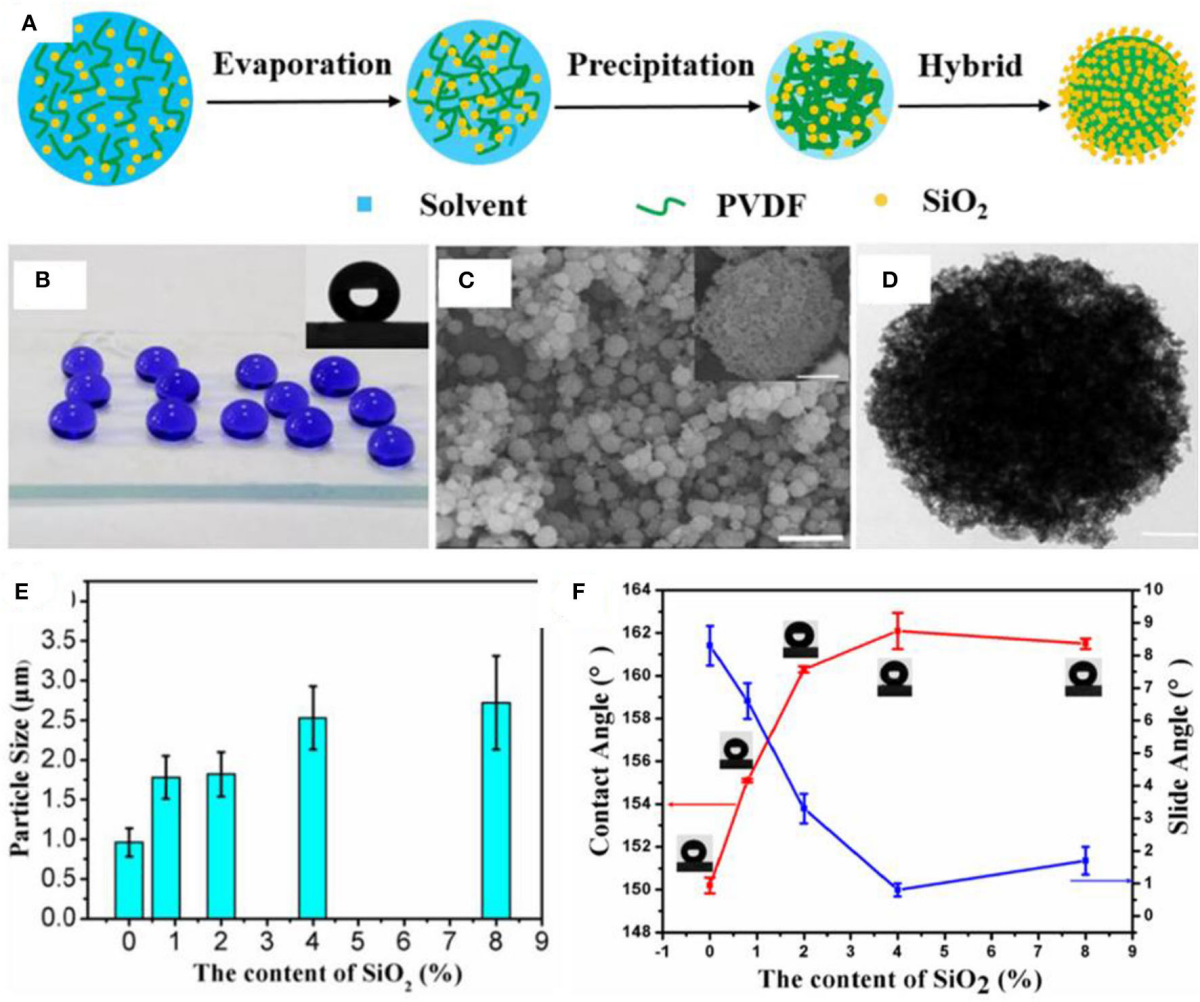
**(A)** Schematic showing the procedure for synthesis of hierarchical hybrid microspheres. **(B)** Digital images showing the water droplets on the superhydrophobic surface, whereas optical photograph in the inset represents contact angle measurement **(C)**. SEM (C, scale bar = 10 and 1 μm for inset) and **(D)** transmission electron microscopy (TEM) images showing the morphology of electrosprayed hybrid microspheres. **(E)** Plot showing the change in diameter of hybrid particles as a function of silica concentration. **(F)** The graph shows the effect of silica content on contact angles and sliding angles of water drops. Adapted from Gao et al. (2017a). With permission from Elsevier, copyright 2017.

In another study, core–shell particles were produced by deposition of silica on the surface of polyvinylidene fluoride (PVDF) microspheres with hierarchical microscale and nanoscale roughness (Figure 6A) (Gao et al., 2017a). Silane-modified silica nanoparticles and PVDF were mixed in an organic solvent followed by electrospraying, which resulted in precipitation of PVDF and evaporation of solvent. At an appropriate concentration of silica, that is, 8%, a core shell structure was synthesized where PVDF particles acted as an organic core while silica nanoparticles formed a continuous inorganic shell (Figures 6C,D).

The size of PVDF particles increased gradually with increasing amount of silica (Figure 6E), which is probably due to an increase in thickness of shell. Surface coated silica plays an important role in dictating the superhydrophobic properties as evidenced by the increasing contact angle with increasing amount of silica (Figure 6F). This behavior was attributed to the increased roughness with increasing content of silica. In addition to the superhdyrophobic coating by electrospraying (Figure 6B), a free-standing membrane was formed by using the same materials; however, the membrane demonstrated low WCA as compared with the superhydrophobic coating.

**Figure 7:**
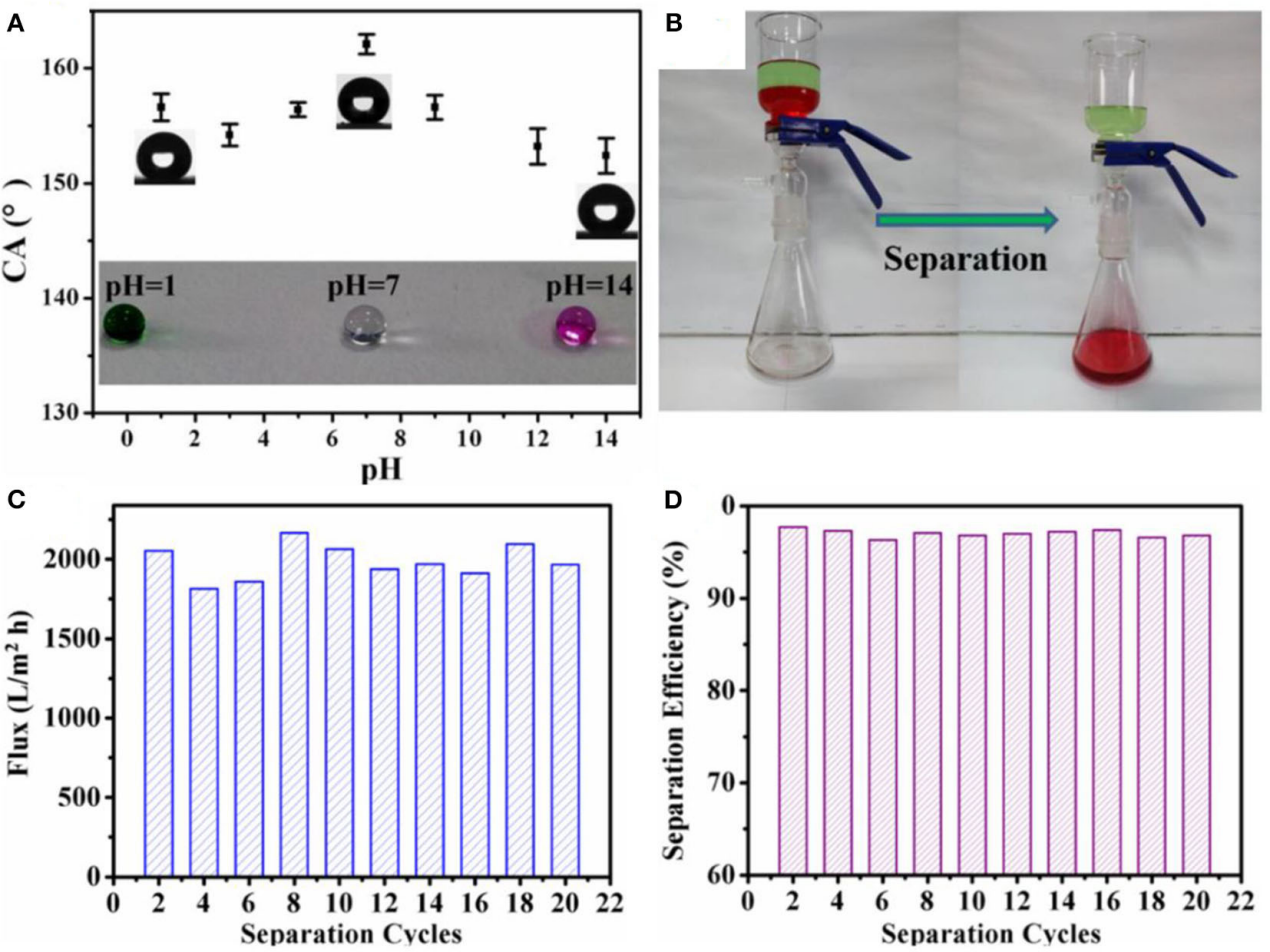
**(A)** Graph showing the variation of water contact angles at different pH values, whereas the shape of water droplets can be observed in the inset. **(B)** Separation of dichloromethane and water under harsh conditions. The **(C)** flux and **(D)** efficiency for the oil-water separation with different cycles by using the polyvinylidene fluoride (PVDF)/silica membrane. Adapted from Gao et al. (2017a). With permission from Elsevier, copyright 2017.

The superhydrophobic coating was applied on filter paper to demonstrate its application in oil/water separations. The coated filter paper proved to be effective in the separation of oil/water mixtures and also resisted corrosive liquids (Figures 7A,B). However, the filter paper took 3 min to separate 100 ml of a mixture. On the other hand, a membrane formed by electrospraying PVDF and silica took only 30 s to separate the same volume. It was suggested that the superior properties of the membrane were due to superoleophilic properties, and the capillary tubes formed by voids between the microspheres and threads resulted in quick and continuous transportation of oil across the membrane during the separation process. The increased separation time in the case of modified filter paper was attributed to the poor transportation of oil through the structure of filter paper. The membrane separated an oil/water mixture with flux of 2,050 L m^−2^ h^−1^ and separation efficiency of 97.5% up to 20 cycles (Figures 7C,D).

#### Nanostructured Scaffolds

Nanostructured scaffolds of silica have been fabricated and used for the absorption and removal of oil from oil/water mixtures. In comparison with particles, they are interconnected networks and offer multiple advantages such as recyclability, easy handling, and good absorption.

**Figure 8:**
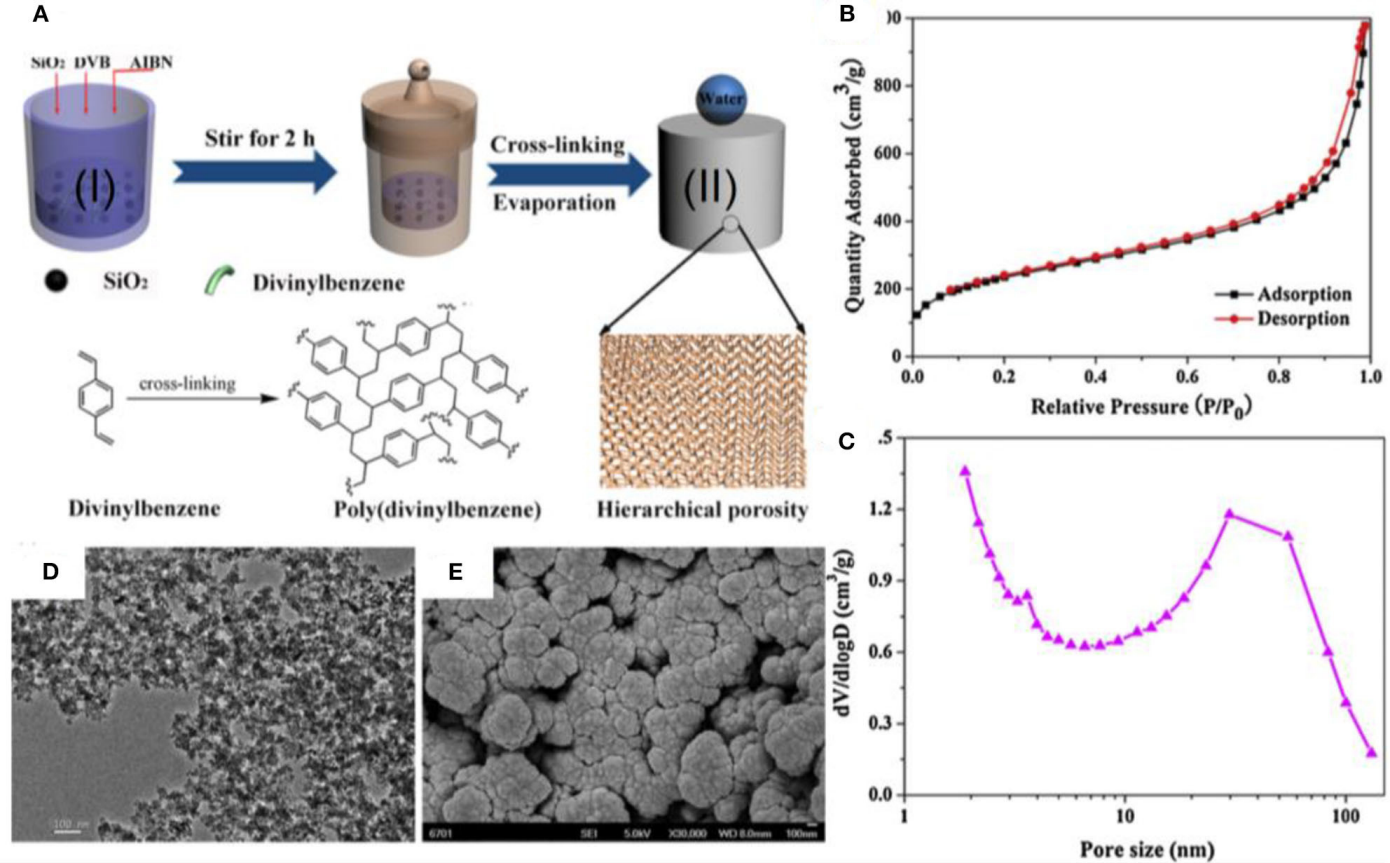
**(A)** Schematic showing the fabrication process of hybrid material. **(B)** Gas adsorption isotherms and **(C)** pore size distribution confirm the nanoporous structure of hybrid material. **(D)** Transmission electron microscopy (TEM) images **(E)** and SEM images of hybrid material. Adapted from Li et al. (2017c). With permission from Elsevier, copyright 2017.

Nanoporous hybrid materials have been fabricated by a solvothermal process from divinylbenzene (DVB) and silica for the separation of different types of emulsions (Li et al., 2017c). The fabrication procedure involves the reaction of fumed silica (0.2–0.3 μm) with DVB monomer in the presence of radical initiator azobisisobutyronitrile (AIBN) (Figure 8A). The interconnected porous material that results from this reaction was isolated by evaporating the ethyl acetate.

Characterization of the surface by Brunauer–Emmett–Teller (BET) adsorption isotherms and SEM revealed mesopores with diameters from 10 to 100 nm (Figures 8B,C). SEM and TEM revealed an overall hierarchical structure constituted by different types of nanoparticles (Figures 8D,E). This unique hierarchical structure provides air pockets during interaction with liquid droplets and renders the material superhydrophobic.

Indeed, the prepared material demonstrated a WCA of 160° and complete repellence of water. Owing to excellent superhydrophobic properties and selective wetting toward oil due to superoleophilic properties, the material immediately adsorbed the hexadecane from a water/hexadecane mixture. Different types of common organic pollutants were separated, and the respective absorbance capacity was reported. It was found that the absorbance of the material is as high as 21.9 g g^−1^.

**Figure 9:**
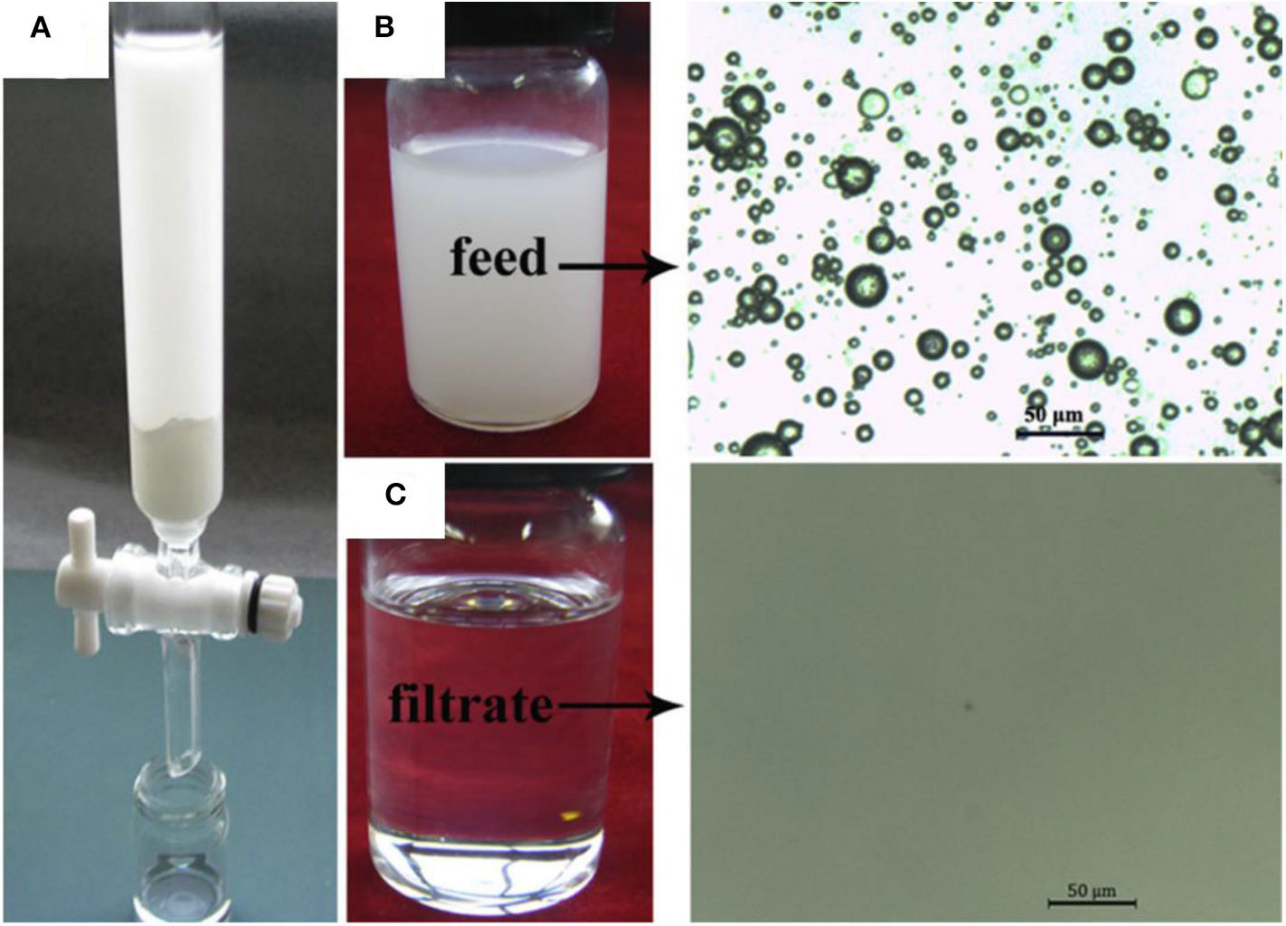
**(A)** Digital images showing the setup for separation of water-in-oil emulsion. Optical microscope results of water in toluene emulsion before **(B)** and after **(C)** separation. Adapted from Li et al. (2017c). With permission from Elsevier, copyright 2017.

The material was also tested for separation of surfactant stabilized oil in water or water-in-oil emulsions (Figure 9A). These emulsions were prepared by mixing the fluids in 1:100 ratios, stabilizing them with the commercially available non-ionic surfactants Tween 80 or Span 80 with stirring for 15 h. In the case of a water in toluene emulsion, the toluene easily passed through the porous channels, whereas water was completely rejected owing to the superhydrophobic properties of the membrane. It was further confirmed from optical microscopy that de-emulsification took place, as effectively indicated by the significant difference in the phase contrast observed before and after separation (Figures 9B,C). Moreover, dynamic light scattering (DLS) measurements also verified the substantial change in droplet sizes before and after separation of emulsion. It was confirmed by gas chromatography that the separated toluene was 99.73% pure, which proved the high separation efficiency of the process.

### Carbon

Elemental carbon is an important element that exists in both crystalline (diamond and graphite) and non-crystalline bulk allotropes (coal, charcoal, and carbon black). It contains a total of six electrons with four in its valence electrons with three different hybridizations: *sp*^3^, *sp*^2^, and *sp*. The 1s^2^, 2s^2^, and 2p^2^ electronic configuration of carbon allows it to have several types of structures with unique properties. For example, diamond is the hardest material, as every carbon atom is covalently bonded to four other carbon atoms in tetrahedral fashion, whereas graphite is a soft material, as it consists of 2D sheets of carbon atoms joined by Van der Waals forces. The weak bonding (ca. 2 kJ mol^−1^) between the sheets allows sliding, which makes graphite a soft material. The carbon atoms in graphene sheets are, however, bonded via covalent bonds. The nanoscale allotropes of carbon including graphene, carbon nanotubes (CNTs), fullerenes, and nanodiamonds offer a wide range of properties such as high thermal and electrical conductivity, corrosion resistance, optical, mechanical, and electrochemical properties (Zhai et al., 2017). The use of carbon-based nanomaterials has been extended to structural materials, electronic, sensing, biomedical, energy storage, catalysis, cardiac scaffolds, desalination, and water purification (Zhai et al., 2017; Chen et al., 2020; Liu et al., 2020; Zhang et al., 2020a). Different forms of carbon have been explored to prepare materials for separation of oil/water mixtures and emulsions. Carbon foam fabricated by using PU foam as a template and lignin phenol formaldehyde as a carbon source is just one example of carbon-based materials for oil/water separation (Qu et al., 2017). Several other forms of carbon have also been used for this purpose and are discussed in detail in the following sections.

#### Graphene

Graphene is a single layer formed by hexagonally arranged *sp*^2^-hybridized carbon atoms. Graphene exhibits interesting properties such as very-high electron mobility (250,000 cm^2^ V·s^−1^ at room temperature), high Young's modulus (1 TPa) and thermal conductivity of 5,000 W m^−1^ K^−1^ (Singh et al., 2011; Randviir et al., 2014). Graphene has been utilized to produce materials for oil/water separation (Alammar et al., 2020; Dai et al., 2020; Tang et al., 2020). It has strong tendency toward absorption of oils, and this property was employed to create a superhydrophobic foam by dip coating a PU sponge in suspensions of graphene and cellulose nanowhiskers. The superhydrophobic foam displayed effective separation of several organic solvents and maintained its superhydrophobicity after 50 cycles of separations (Zhang et al., 2017d). In another example, graphene was introduced in the framework of a PU sponge by *in situ* polymerization in the presence of *N*-methylpyrrolidone (Kong et al., 2017). The resulting sponge showed a WCA of 151.8 ± 0.5° and an oil contact angle of 0.5° and was subsequently used for the separation of various types of oil/water mixtures and emulsions.

Magnetic GO sheets were synthesized for the separation of oil/water emulsions (Liu et al., 2017a). Magnetic ferrite particles were prepared from iron carbonyls by oxidation/decomposition method followed by a thin coating of silica to enhance the chemical stability and to provide a medium for functionalization with silane coupling agent (3-aminopropyl)triethoxysilane (3-APTES). The amine groups on the surface were then utilized for the reaction with GO sheets prepared by Hummer's method (Supplementary Information). Separation experiments were performed by using a crude oil-in-water emulsion, which was stable up to 12 h. A small quantity of M-GO was introduced in the mixture, which immediately changed its color, indicating the breaking of the emulsion (Supplementary Information).

**Figure 10:**
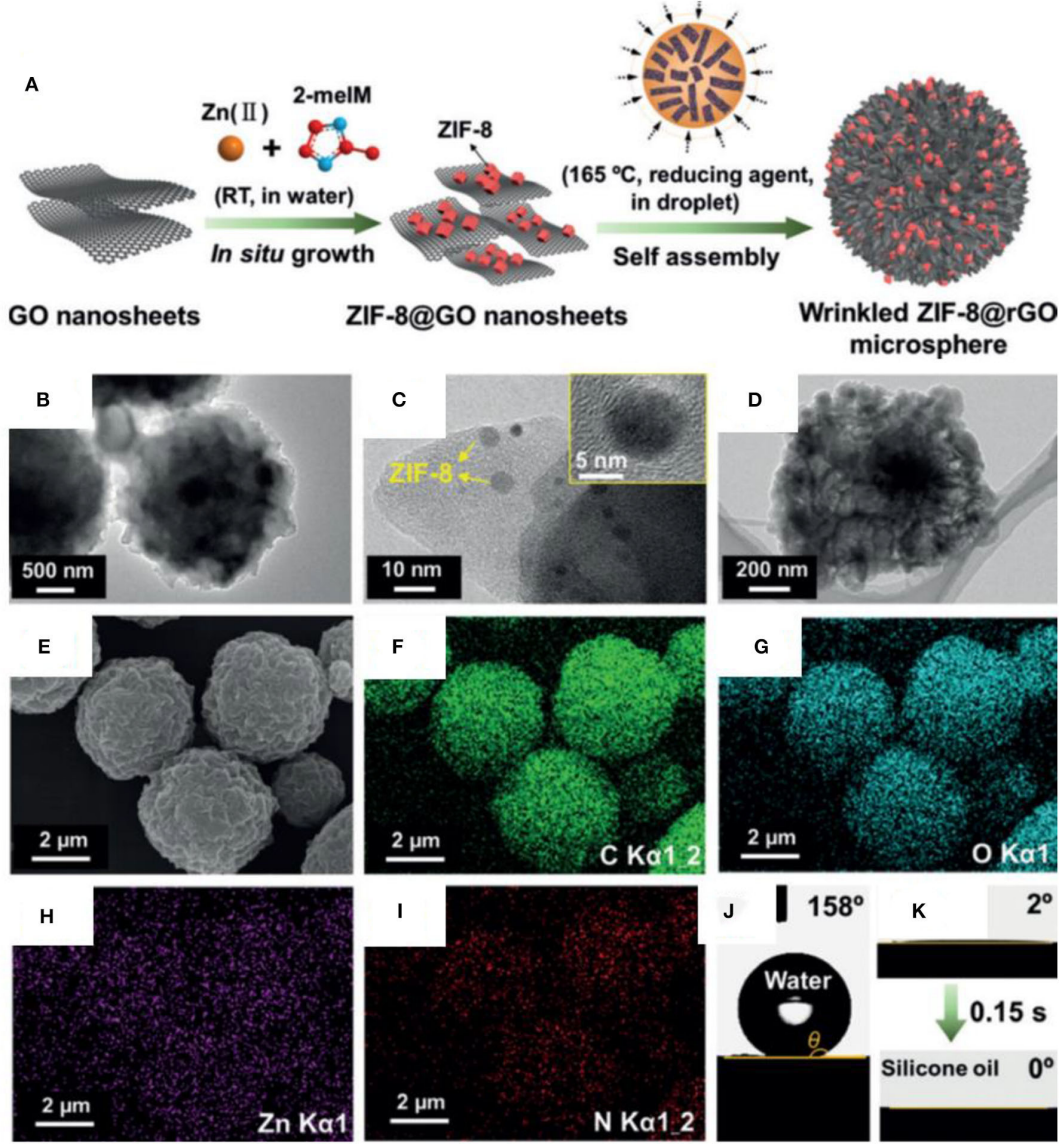
**(A)** Schematic showing the formation process of ZIF-8@rGO spheres. **(B–D)** Transmission electron microscopy (TEM) images showing the structure of the wrinkled ZIF-8@rGO. **(E)** SEM and **(F–I)** energy-dispersive spectrometry (EDS) of ZIF-8@rGO. **(J)** Contact angle of water and **(K)** silicone oil on ZIF-8@rGO (Gu et al., 2019).

Recently, reduced GO-based composites with a hierarchical structure have been fabricated by embedding metal organic framework nanoparticles in rGO sheets (Gu et al., 2019). The material possesses a combination of superhydrophobic and superoleophilic properties, allowing it to selectively capture oil from an oil/water mixture. The composite was fabricated by immobilizing zeolitic imidazolate frameworks (ZIF-8) on rGO sheets at room temperature. The microdroplets of suspension of composite were produced by using an ultrasonic spray nozzle. The droplets were mixed with silicon oil (continuous stirring at 165°C) to allow the wrinkling and self-assembly of composite microspheres. Figure 10A shows the schematic of synthesis process.

Figures 10B–D show the TEM images of ZIF-8@rGO microparticles. These are composed of ZIF-8 nanoparticles uniformly distributed on wrinkled rGO sheets. The spatial uniformity and dispersion of ZIF-8 can be observed from the energy-dispersive X-ray (EDX) (Figures 10E–I). The combination of nanoparticles and sheet structure creates mesoscale roughness, which helps to achieve very-high contact angle of 158° along with oleophilic properties (Figures 10J,K). The produced particles were coated on a PU foam by dip coating, and the resulting composite superhydrophobic foam was used for the separation of organic solvents from water.

#### Carbon Nanotubes/Fibers

CNTs can be imagined as a cylinder in which individual carbon atoms are *sp*^2^ bonded to each other. There are different types of CNTs including single wall, double wall, or multiwall, depending upon the number of rolled graphene sheets. They have very-high surface area and can be easily functionalized both covalently and non-covalently to host new properties for specific applications. CNTs can be chemically modified by carboxylation, amidation, acrylation, and PEGylation and esterification. Another interesting advantage of CNT is their dual-scale roughness due to the cylindrical structures. This feature is very important in fabrication of superhydrophobic materials (Yang et al., 2020a; Zarghami et al., 2020). A composite membrane for the separation of oil/water emulsion was fabricated by using CNTs and polysulfone as a support. A thin layer of pebax was coated, and different concentrations of functionalized CNTs (FCNTs) were used to study the effect on morphology and separation characteristic of the membrane (Saadati and Pakizeh, 2017). It was observed that the increasing concentration of FCNTs improved the mechanical and thermal properties and increased the hydrophilicity of membrane. Silanized CNTs have been used to fabricate ethyl cellulose-based composite sponges. The sponges were coated by nanoparticles of SiO_2_, which were further modified with hexadecyltrimethoxysilane to finally obtain a superhydrophobic and superoleophilic sponge. The resulting sponge was then used for the separation of oil/water mixtures and emulsions (Lu and Yuan, 2017). In another study, a superhydrophobic and superoleophilic membrane was prepared by assembling carbon nanofibers and single-walled CNTs on an etched stainless steel mesh. The composite membrane was then modified with polydimethysiloxane and used for the separation of water from an emulsion (Lin et al., 2017).

Superhydrophobic surfaces have been fabricated via two step fabrication of carbon nanofibers by plasma sputtering followed by chemical vapor deposition (CVD) at 300°C to achieve superhdyrophobic properties (Siddiqui et al., 2017). This represents a route that does not involve complicated processing and avoids toxic chemicals. Initially, the copper was deposited on different substrates as a catalyst by plasma sputtering. Later, the samples were placed in a CVD chamber for carbon deposition. The flow of hydrogen was maintained until the temperature reached 300°C, and then acetylene was introduced for 30 min. Finally, the chamber was cooled to room temperature under nitrogen.

**Figure 11:**
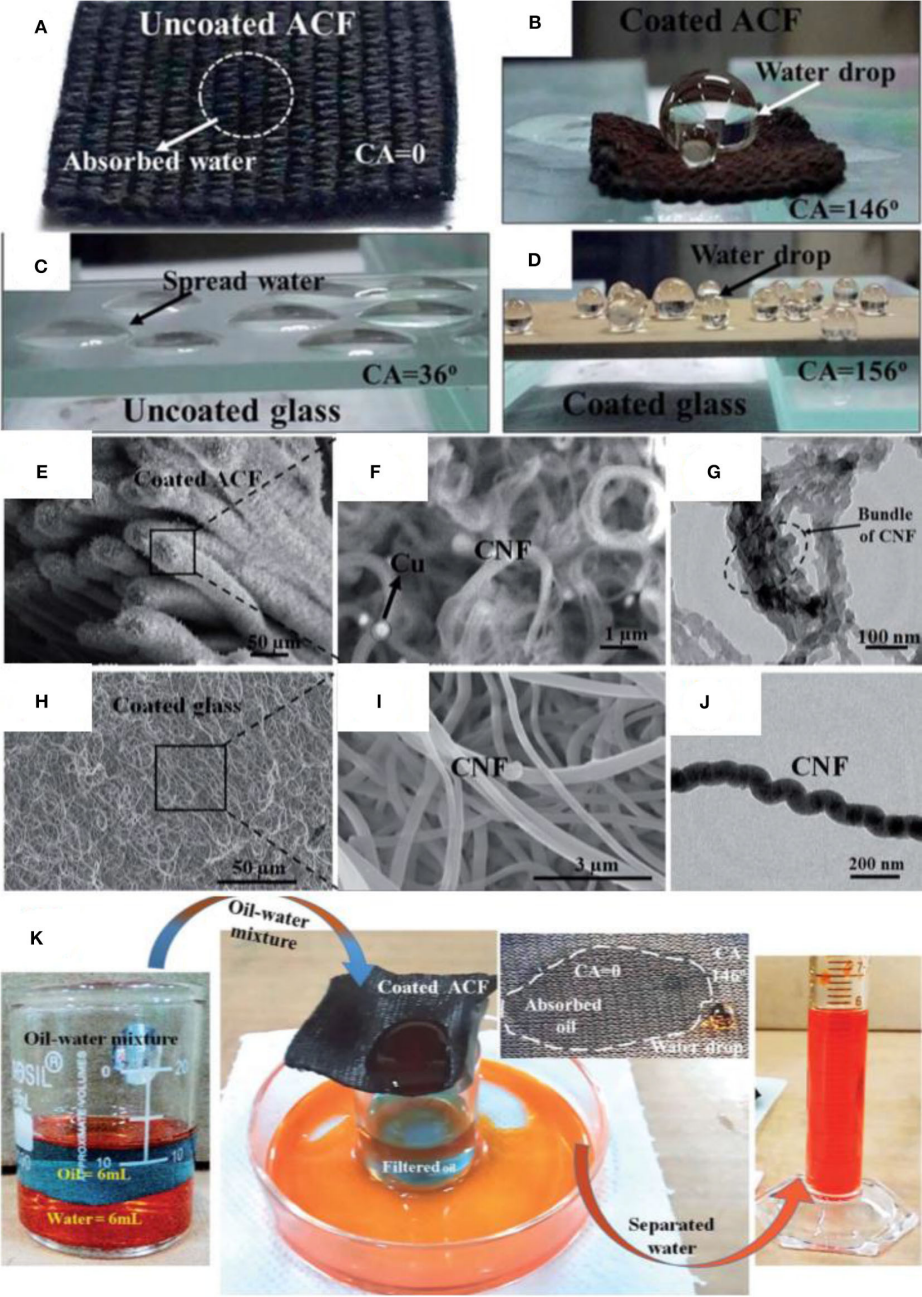
**(A)** Digital images showing pristine ACF **(B)** and modified ACF **(C)** and pristine glass **(D)** and modified glass **(E–J)**. SEM and transmission electron microscopy (TEM) images of modified ACF and glass. **(K)** Separation of kerosene and water mixture by using modified ACF. Adapted from Siddiqui et al. (2017). With permission from the Royal Society of Chemistry, copyright 2017.

Digital images show the wetting behavior of glass and fabric before and after deposition of CNF (Figures 11A,B), and it can be observed that the water drop has formed a bead shape on superhdyrophobic materials as compared with complete absorption or very low contact angle in the case of pristine materials.

SEM and TEM results reveal the presence of entangled CNF structure with individual fiber with a diameter of 30 nm, whereas the Cu particles have been observed as bright spots (Figures 11E–G). Similarly, CNF structure on glass can also be seen with an individual fiber of diameter of <100 nm (Figures 11H–J). The porous structure formed by CNF is supposed to entrap more air, thus increasing surface superhydrophobicity (Siddiqui et al., 2017).

The pristine fabric shows the water contact angle of 0°; however, the deposition of CNF increased the water contact angle to 146°. Similarly, CNF-coated glass also demonstrated an increase in water contact angle from 36° to 156°. Surface energy measurements showed that the coated glass slide and fabric (46.56 and 55.91 mJ m^−2^, respectively) hold low surface energy as compared with the uncoated glass and fabric. XPS showed that the coated glass slide and fabric contained less carbonyl groups as compared with the uncoated samples. The coated activated carbon fiber (ACF) has a combination of superhydrophobic and superoleophilic behaviors, which means that oil can easily pass through it while water would be completely rejected (Figure 11K). The property was used to separate different types of oil/water mixtures with a separation efficiency of 99% up to 50 cycles (Siddiqui et al., 2017).

#### Carbon Soot

Carbon nanoparticles (CNPs; soot) are produced form incomplete combustion of organic molecules. One simple source of CNPs is candles, which are primarily made of paraffin wax. Incomplete combustion of candle wax results in the production of hydrophobic CNPs. Despite some limitations in terms of stability and mechanical properties, it is an inexpensive and simple process to fabricate superhydrophobic materials. Candle soot has been used to produce superhydrophobic and superamphiphobic surfaces while using polydimethylsiloxane (PDMS) and hexane as base (Iqbal et al., 2017). CNPs were embedded in PDMS, which was cured by heat of the flame. This resulted in chemically and mechanically stable superhydrophobic surface. Similarly, a robust superhydrophobic coating was produced by depositing a composite coating of camphor soot and PDMS (Sahoo et al., 2017). In another study, CNPs dispersed in acetone were prepared by using a candle (Qahtan et al., 2017). The dispersion was then used to prepare superhydrophobic coatings by spray process. The coatings produced by this method were stable up to 400°C and demonstrated better water jet and drop impact resistance than did the coatings prepared by direct deposition of soot from candle flame.

**Figure 12:**
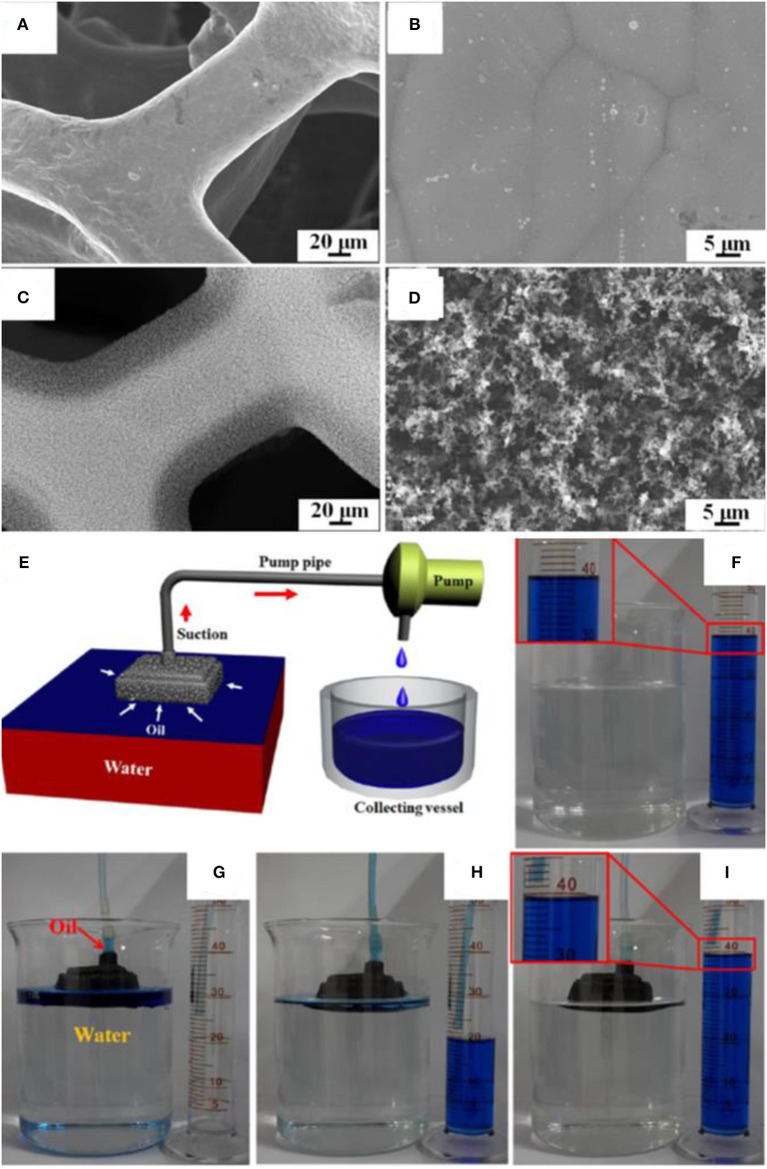
**(A,B)** SEM images of copper foam before and **(C,D)** after coating with candle soot. **(E)** Schematic showing the separation apparatus. **(F–I)** Digital images showing the separation of oil/water mixtures by using superhydrophobic copper foam. Adapted from Ju et al. (2017). With permission from the American Chemical Society, copyright 2017.

A one-step method was proposed to fabricate superhydrophobic and superoleophilic materials (Ju et al., 2017). The proposed fabrication method does not require any chemicals, and short smoking for 5 min was enough to render the materials superhydrophobic. A folded copper foam was exposed to the flame of candle under suitable conditions to collect the carbon soot. The deposition of carbon soot created hierarchical structures with microscale/nanoscale features, which are absent in the bare copper foam as shown in the SEM images (Figures 12A–D).

SEM, XPS, and EDS results confirmed the presence of a 20-μm-thick layer of carbon soot, which wrapped the porous structure of copper foam. The hierarchical structure together with low surface energy of carbon soot realized the superhydrophobic coating without any chemical modification. Contrary to the superhydrophilic behavior of pristine copper foam, it demonstrated water contact angle of 155° after coating with carbon soot. Moreover, the superhydrophobic copper foam demonstrated superoleophilic behavior as the oil droplet completely spread over it. The copper foam was used for the gravity-driven separation of an oil/water mixture containing 15 ml of red-dyed water and 30 ml of blue-dyed toluene. The oil could easily pass through the foam while water was retained in the foam, resulting in the complete separation of an oil/water mixture (Qahtan et al., 2017).

In addition to gravity separation, continuous separation was performed from a device containing a peristaltic pump as shown in Figure 12E. The device efficiently separated the oil from an oil/water mixture containing 450 ml of water and 40 ml of toluene dyed with methylene blue (Figures 12F–I). The device separated the mixture with a recovery rate of 98.8%, which was based on the comparison of gathered oil and the original amount in the mixture (Qahtan et al., 2017).

Following this approach, five different oil/water mixtures of toluene, *n*-hexane, silicone oil, isobutanol, and dichloromethane were separated; and their recycled rates (volume ratio of the collected oil and original amount of oil in a mixture) were calculated. Moreover, the device demonstrated recovery rate of more than 95% up to six cycles, ensuing the reproducibility and durability.

#### Carbon Gels

Aerogels are known for their porous structure and very low density. A typical synthesis approach for aerogels is to prepare wetgel by a sol–gel process followed by replacing the liquid phase with gas. The aerogels have high porosity, which give them distinct properties including high surface area and very low thermal conductivity. As the porous structure of carbon aerogels is assembled from *sp*^2^ CNPs, they possess unique properties such as high thermal, mechanical, and chemical stability; electrical conductivity; large surface area; and very low density. These fascinating characteristics persuaded the researchers to find the application of carbon aerogels in many fields such as sorbents, energy, filtration, and sensors (Chandrasekaran et al., 2017). Carbon-based aerogels can be divided into five different types on the basis of the allotrope of carbon used in the production of aerogel (Chandrasekaran et al., 2017). For example, GO has been used to prepare graphene-based aerogels (Gorgolis and Galiotis, 2017). GO can be suspended in water and contains oxygen functionalities at basal planes and edges, which helps in covalent reaction with different compounds. This ability allows the preparations of prepare new materials with tunable properties for specific applications. Similarly, CNTs have been used to prepare robust and conductive aerogels for a range of energy and environment applications (Lin et al., 2016). Other examples of carbon aerogels are amorphous carbon, graphite, and diamond aerogels (Pauzauskie et al., 2011; Maleki, 2016; Chandrasekaran et al., 2017).

Different types of carbon-based aerogels and their composites have been conceived for oil/water separation. For example, a PU foam-reinforced spongy graphene aerogel was fabricated by freeze casting for collection of oil from an oil/water mixture (Luo et al., 2017). The produced aerogel demonstrated superior mechanical and superhydrophobic properties, making it applicable for separation of oil/water mixtures for several cycles. In another study, lightweight and electrically conductive carbon aerogels were fabricated from oxidation-oven drying-carbonization method by using waste paper as a source material (Li et al., 2017b). The aerogel produced by this method is suitable for separation of oil/water mixtures owing to the combination of superhydrophobic/superoleophilic properties, high absorption (33–70 g g^−1^), and high compressibility. In a different study, popcorn was used as carbon precursor to prepare superhydrophobic and magnetic carbon aerogel (Dai et al., 2018). The popcorn was treated with the iron nitrate prior to carbonization to yield magnetic popcorn carbon, which was further modified with octyltrichlorosilane to produce “superhydrophobic aerogels.” The aerogel exhibits selective absorption toward oils and can be used for separation for many types of oil/water mixtures up to several cycles. Similarly, pressure-sensitive carbon aerogels were fabricated from pyrolysis of cellulose aerogels, which were composed of microfibers of poplar catkin (PC) (Li et al., 2017a). For this, the PC fibers were activated by sodium chlorite followed by carbonization at 1,000°C under nitrogen atmosphere (more details in the Supplementary Information).

**Figure 13:**
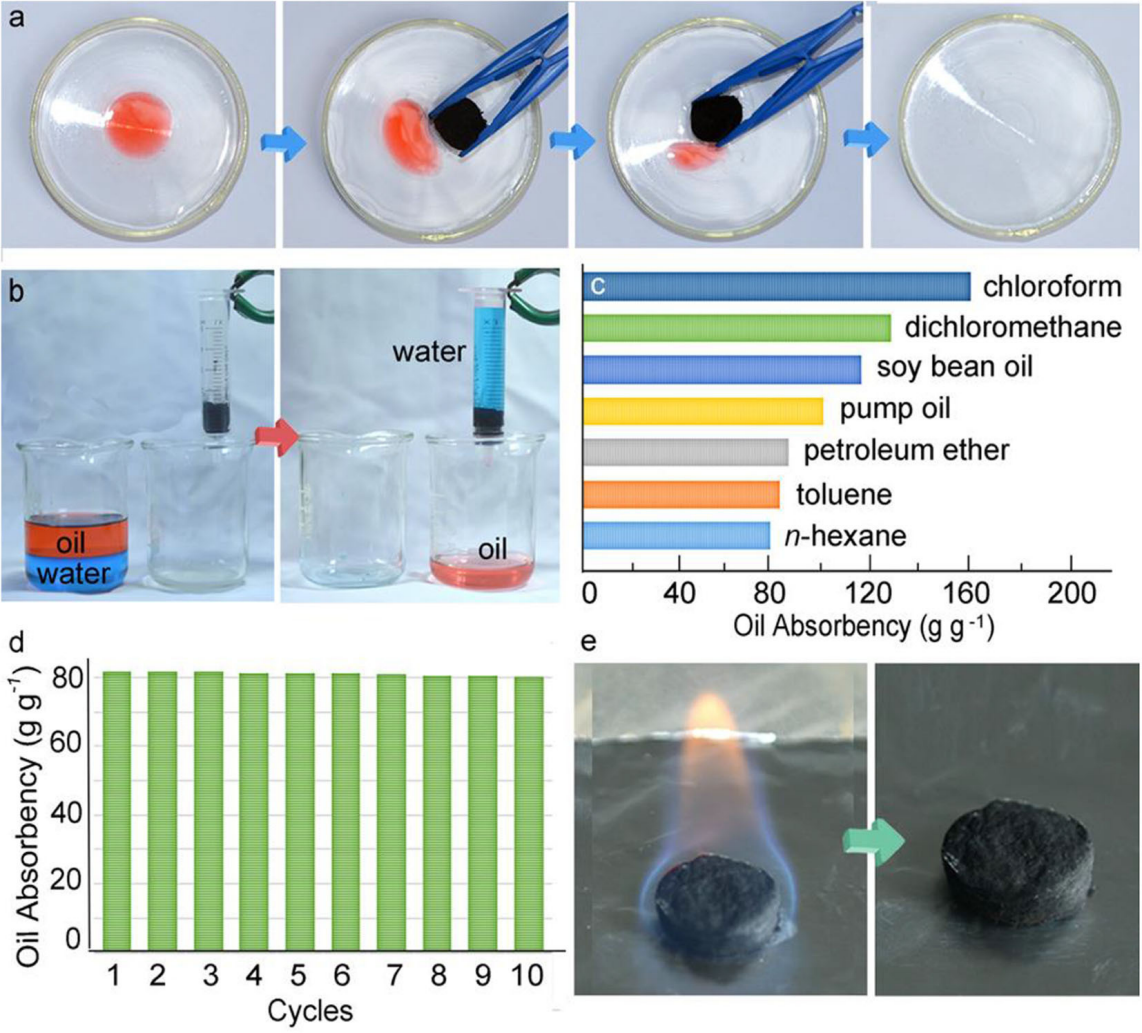
Digital images showing the **(a)** absorption of oil **(b)** separation process, **(c)** absorption capacity, **(d)** cycles, **(e)** combustion of oil in pressure-sensitive and (PSC) gel. Adapted from Li et al. (2017a). With permission from the American Chemical Society, copyright 2017.

The pressure-sensitive and conductive (PSC) aerogels have a water contact angle of 150.3°, making them suitable candidates for the absorption of the separation of oil/water mixtures (Figure 13a). The PSC aerogel can separate oil/water mixtures under gravity as shown in Figure 13b. The oil was passed through the aerogel while water was retained on the surface. The aerogel displayed an absorbency of 81–161 g g^−1^ for a range of oils and organic solvents as shown in Figure 13c. Moreover, the aerogel can be reused after drying in an oven and displayed absorbance 80 g g^−1^ when used for 10 cycles. It was demonstrated that the oil-absorbed aerogel can also be treated via combustion (Figures 13d,e). The aerogel maintained its skeleton after complete combustion of oil in 45 s.

### Iron Oxide

Iron oxides naturally exist in many forms; however, magnetite, (Fe_3_O_4_), maghemite (γ-Fe_2_O_3_), and hematite (α-Fe_2_O_3_) are technologically the most important types. These oxides have found applications in many fields owing to their magnetic properties and biocompatibility. Magnetite (Fe_3_O_4_) and maghemite (γ-Fe_2_O_3_) have been explored extensively for biomedical applications including drug delivery, thermal therapy, magnetic hyperthermia, and magnetic resonance imaging (Kandasamy and Maity, 2015; Lee et al., 2015; Ali et al., 2016; Mirabello et al., 2016), whereas hematite (α-Fe_2_O_3_) has been used in gas sensors, pigments, and catalysts, as it is an n-type semiconductor, has low cost, and shows resistance against corrosion (Wu et al., 2016).

Inspired by its magnetic properties, Fe_3_O_4_ has been used in various studies alone or with other materials for separation of oil/water mixtures and emulsions (Su et al., 2016; Lü et al., 2017; Zhang et al., 2017c; Guselnikova et al., 2020). For example, Fe_3_O_4_ was deposited on coco peat powder with the aid of polydopamine followed by modification with octadecylamine. The resulting product was magnetic and highly hydrophobic at 135 ± 3°. These properties make it suitable for sorption of oil from an oil/water mixture (Yang et al., 2017a). Similarly, Fe_3_O_4_ was used to prepare a magnetic and superhydrophobic PU sponge (Beshkar et al., 2017). The sponge was coated with straw soot and Fe_3_O_4_ nanoparticles via an immersion method. The sponge was later modified with PDMS to improve the water-repellant character of the sponge. The sponge exhibited water contact angle 154° and oil absorption 30 times its own weight. Moreover, the sponge is recyclable up to 30 times and suitable for magnetic separation. In some other studies, Fe_3_O_4_ nanoparticles have been used in combination with other materials such as zinc oxide, stearic acid (Tran and Lee, 2017), silane (Liu et al., 2015; Li et al., 2018c), silica, and fluoropolymer (Wu et al., 2015) to modify a PU sponge for oil/water separation.

Recently, Fe_2_O_3_ nanoparticles prepared by a co-precipitation method were coated with silica and APTES followed by grafting with quaternized chitosan (QC) (Zhang et al., 2017c). The prepared material was then characterized for oil/water separation with respect to PH, reusability, and as a function of dosage. To study the oil/water separation ability of prepared materials, a 0.2-wt.% emulsion of diesel in water was made via sonication for 5 min. A known amount of nanoparticles was added followed by manual shaking and separation of nanoparticles by applying a magnetic field. The nanoparticles were then washed with ethanol three times to remove the oil and to reuse for the separation experiments. The separation experiments were performed under different pH values, and it was found that QC-coated nanoparticles shows exceptional performance. The transmittance of water increased with increasing dosage of QC-coated nanoparticles. The equilibrium value of 98% was achieved when dosage was increased to 34 mg L^−1^ under both acidic and neutral conditions. However, the separation efficiency was decreased under alkaline conditions, and 98% transmittance was achieved. It was discussed that negatively charged oil droplets were attracted toward positively charged QC-coated nanoparticles via electrostatic attraction.

### Titanium Oxide

Titania naturally exists as anatase, rutile, and brookite. It is an n-type semiconductor (3.02 eV) and has very-high refractive index of 2.609 (rutile). When exposed to UV light, it generates exciton, which can be used for the production of electricity or photo catalysis (Theivasanthi, 2017). Titania has been explored for many applications including degradation of organic pollutants, photovoltaics, air purification, self-cleaning surfaces, hydrogen evolution, sterilization, food additive, biomedical, degradation of pesticides, supercapacitors, and lithium batteries (Tudu et al., 2020; Wang et al., 2020a; Yang et al., 2020b). An interesting feature of titania is its superhydrophilic property under UV exposure (Nakata and Fujishima, 2012). Inspired by the properties of titania, an underwater superoleophobic membrane was fabricated by spray depositing titania nanofibers and used for the separation of an oil/water mixture and photocatalytic degradation of organic molecules under UV (Gunatilake and Bandara, 2017). In another study, magnetic titania nanotubes were prepared as a superhydrophobic sorbent for oil. Titania nanotubes were synthesized by a hydrothermal method in the presence of magnetic nanoparticles and modified with octadecylamine (Patowary et al., 2017). The prepared material was able to absorb oil more than 1.5 times its own weight and is recyclable up to more than five times. Similarly, a TiO_2_-based superhydrophobic coating was constructed on cotton fabric via a hot pressing technique. The coating had a water contact angle more than 150° and showed resistance to abrasion and machine washing (Gao et al., 2017c). The coating offered self-cleaning, antifouling, and UV-protection properties. Coating with superhydrophobic characteristic was created by dip coating the fabric in a mixture of *n*-octyltriethoxysilane and tetrabutyl titanate. The treatment caused the embedding of randomly dispersed TiO_2_ on the surface of the cotton fibers. The surface roughness was further evaluated by atomic force microscopy, and it was found that pristine fabric contained a relatively smooth structure with root mean square value of 14.6 nm as compared with the coated fabric, which exhibited an increase value of root mean square (RMS) roughness, that is, 48.2 nm. This highlighted the increased roughness of fabrics after deposition of TiO_2_. The inherent structure of fabric combined with TiO_2_ generated a hierarchical structure with microscale and nanoscale features, which are required for the Cassie–Baxter wetting state. A contact angle >150° was observed. The XPS survey of the fabrics confirmed the deposition of TiO_2_ and octyltriethoxysilane as the strong peaks of Ti 2p, Si 2p peak, and Si 2 s were present in the coated fabrics as compared with pristine fabric. The fabric demonstrates outstanding UV resistance and mechanical properties, as it can endure several abrasion and laundering cycles. The fabric was demonstrated to separate a mixture of dichloromethane and water with separation efficiency close to 100%. It can be seen that the water, which was dyed as blue, could not penetrate through the fabric owing to superhdyrophobic property, whereas oil easily permeated through the fabric. The fabric maintained this property even after five cycles of separation.

In another study, TiO_2_ was deposited via spin coating on hierarchical polylactide (PLA) membrane to make it superhydrophilic (Xiong et al., 2017). The PLA membrane was synthesized by a non-solvent induced phase separation (NIPS) method by using a non-woven fabric as a support. The PLA solution was cast on the fabric followed by immersion in water. The membrane was dried in air and peeled off from the fabric. A paint-like suspension of TiO_2_ and as synthesized hydrophilic copolymer P(VP-VTES) was deposited on PLA membrane via spin coating for the fabrication of superhydrophilic surface.

**Figure 14:**
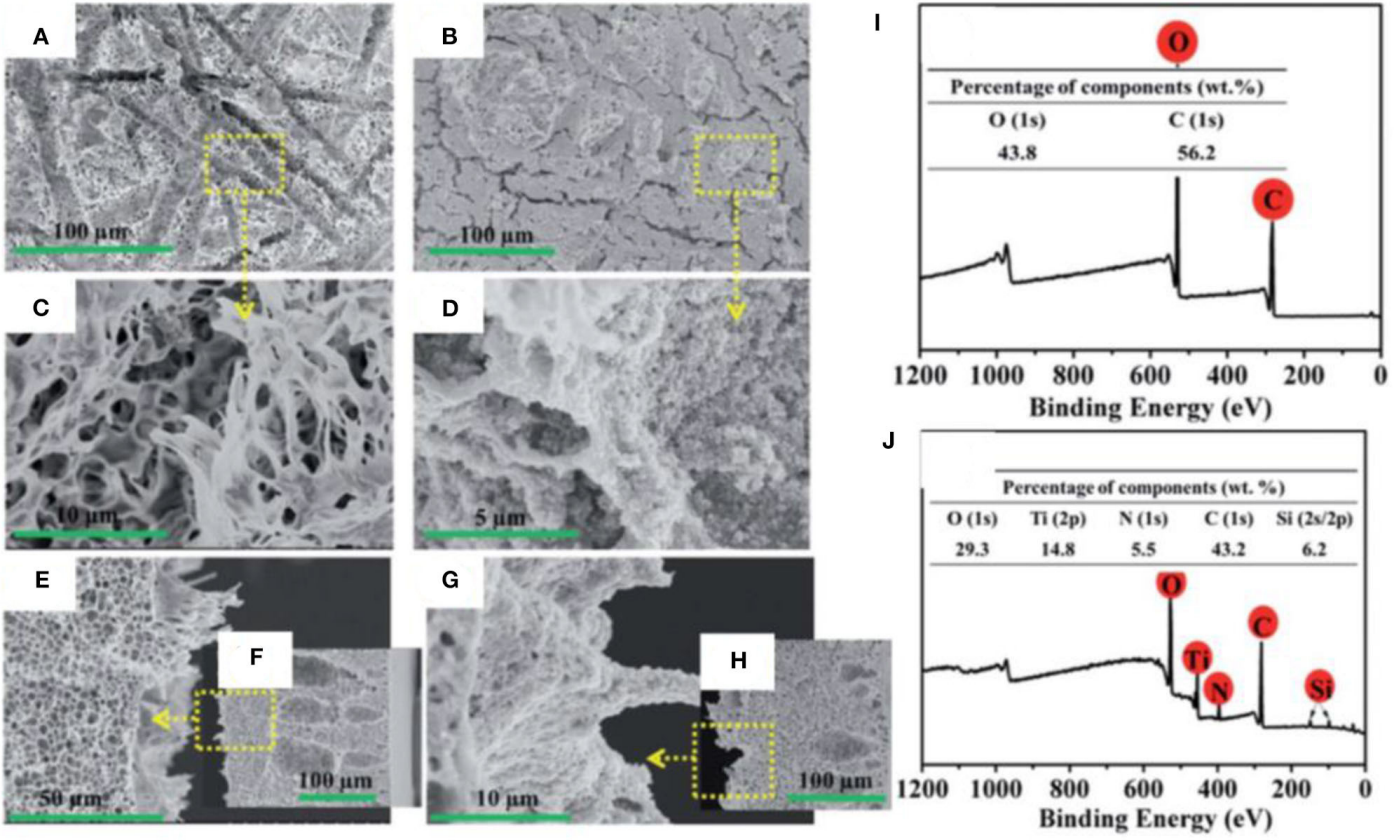
Plan view and cross-sectional SEM images of **(A,C,E,F)** pristine and **(B,D,G,H)** TiO_2_-modified membrane. X-ray photoelectron spectroscopy (XPS) of **(I)** pristine and **(J)** TiO_2_ membranes. With permission from the Royal Society of Chemistry, copyright 2017 (Xiong et al., 2017).

SEM images reveal the morphology of the PLA membrane, and it can be observed that microscale groves, microspores, and stretched nanofibrils have created a hierarchical microstructure. A robust coating of TiO2@P(VP-VTES) was formed on the PLA membrane via spin coating. The TiO_2_ nanoparticles were seized by the stretched nanofibrils to form a coral tentacle-like structure (Figures 14A–H). The high-magnification images show that the TiO_2_ nanoparticles have completely covered the surface (Figure 14G). XPS shows that change in chemical composition of surface after coating with TiO_2_ (Figures 14I,J). Membrane maintained high flux, high separation efficiency, and high rejection after 10 separation cycles of oil/water mixtures (Supplementary Information).

### Zinc Oxide

**Figure 15:**
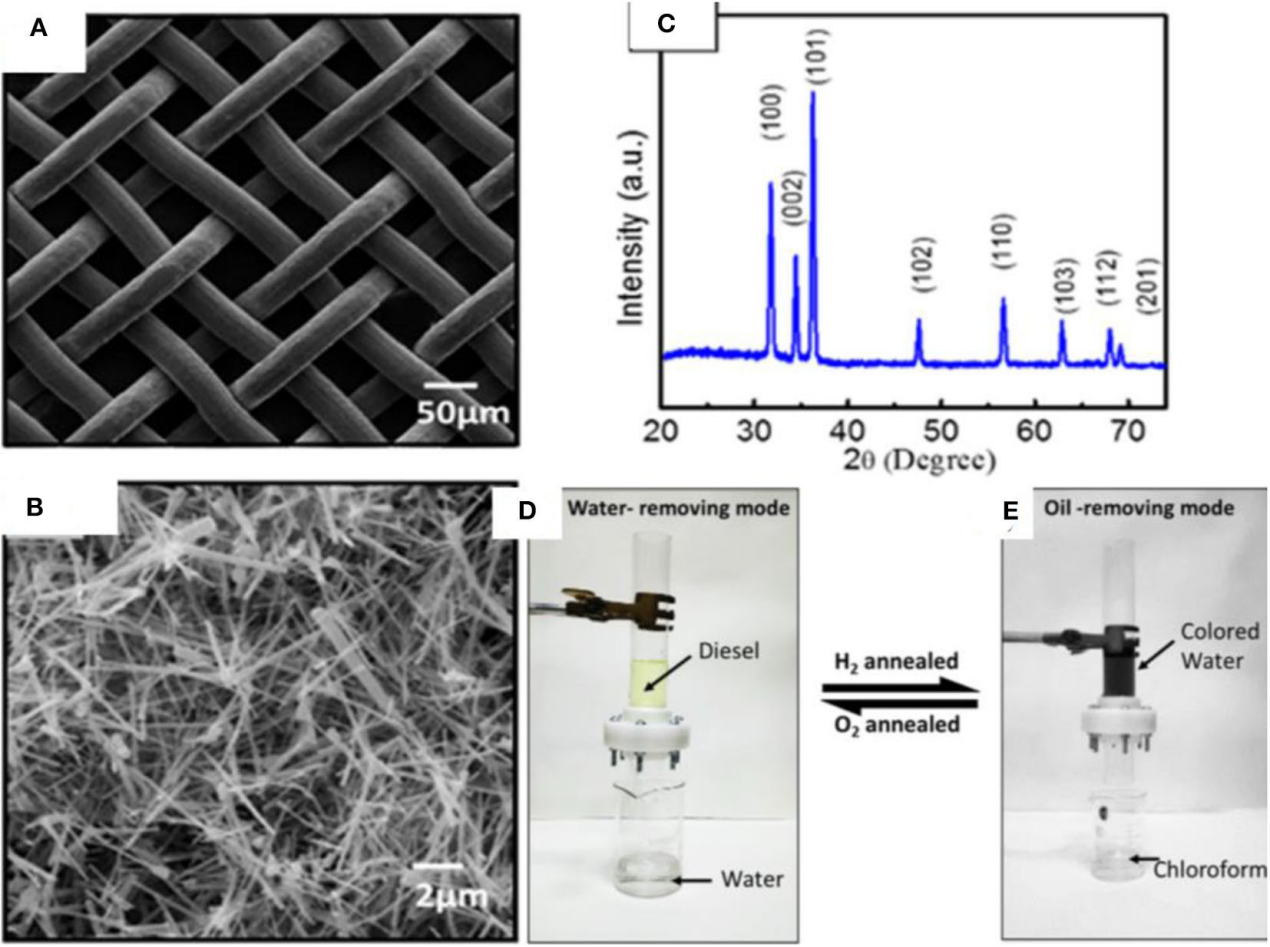
**(A,B)** SEM images showing the structure of pristine and ZnO-coated mesh. **(C)** X-ray diffraction (XRD) pattern of ZnO-coated mesh. **(D,E)** The separation device used for the separation of oil/water mixtures by using ZnO-coated mesh. Adapted from Raturi et al. (2017). With permission from the American Chemical Society, copyright 2017.

ZnO is a semiconductor that has attracted attention owing to its unique physical and chemical properties. ZnO has a large band gap of 3.37 eV, large excitation binding energy of 60 meV, high photo stability, and radiation absorption over a broad range (Agarwal et al., 2017; Ong et al., 2018). Moreover, ZnO is biocompatible and biodegradable and demonstrates antibacterial properties in nano sizes owing to its high surface reactivity (Sirelkhatim et al., 2015). The properties of ZnO have been studied for a wide range of applications including photo catalysis, antibacterial power, energy, sensors, and optoelectronics. A variety of ZnO-based nanostructures have been explored, which can be classified as 1D, 2D, and 3D structures. Some of the examples include nanorods, tubes, needles, belts, rings, sheets, flowers, and snowflakes. Owing to a wide range of nanostructures, ZnO has been used extensively for the fabrication of superhydrophobic surfaces (Huang et al., 2019a; Li et al., 2019a; Lorwanishpaisarn et al., 2019). For example, a superhydrophobic coating was fabricated by using ZnO nanoparticles of size 24 nm and wurtzite structure. The nanoparticles were prepared by a hydrothermal method and modified with palmitic acid. The coating demonstrated a water contact angle >160°; however, it became hydrophilic after heating at 230°. This behavior was associated with the thermal degradation of palmitic acid (Agrawal et al., 2017). Similarly, porous ZnO nanoparticles prepared by a combustion method were used with copper stearate to prepare a superhydrophobic coating with water contact angle 161°. In addition to the nanoparticles, flower-like structures of ZnO have also been explored (Wang et al., 2017b; Zhang et al., 2017e) such as a flower-like ZnO hierarchical structure, which was used in combination with epoxy for durable superhydrophobic surface. The coating demonstrated water contact angle of more than 150° and sustained several cycles of abrasion, demonstrating its high durability. Inspired by the progress in ZnO-based nanomaterials, a unique approach was adopted to develop a mesh for the separation of oil/water mixtures. A mesh was developed by growing ZnO nanowires on stainless steel mesh by CVD, which demonstrated reversible wettability (Raturi et al., 2017). The mesh demonstrated superhydrophilic and underwater superoleophobic behavior, allowing it to let the water penetrate through its pores while blocking the oil. The mesh is able to become superhydrophilic or superhydrophobic, depending upon thermal treatment under hydrogen or oxygen environments. The fabrication route of mesh involved the synthesis of ZnO nanowires produced by thermal CVD, which were then suspended in ethanol and coated on steel mesh by drop casting. SEM images indicate the pore size of mesh (50 μm) and ZnO wires of diameter 100 nm and length 4.6 μm randomly distributed on the surface (Figures 15A,B). The structural analysis of ZnO wires by XRD indicates the wurtzite phase of ZnO (Figure 15C), as prepared mesh demonstrates both superhydrophilic and superoleophilic properties with a water contact angle of 0°.

The mesh displayed underwater superoleophobic behavior. The underwater contact angle is more than 150° for various types of oils, indicating the high resistance of mesh toward oil penetration in the presence of water. Moreover, the oil rolls off at an angle of 5°, indicating the low adhesion, which is due to the repulsion of polar water toward non-polar oils. The trapped water decreases the effective contact area between oil and solid surface, resulting in triple phase discontinuous line. This, together with surface roughness, results in underwater superoleophobic behavior. The mesh was switched to superhydrophobic and superoleophilic by hydrogen annealing treatment. The annealing was performed under hydrogen at 300°C, which resulted in the water contact angle of 154° without affecting the superoleophilic properties of mesh. The reversible switching of mesh was demonstrated by annealing at 300°C for 1 h under oxygen. The process is reversible, and the wetting behavior is controlled by annealing treatments with hydrogen and oxygen.

A model device was fabricated using ZnO nanowire mesh (Figures 15D,E). In the water removing mode, superhydrophilic and underwater superoleophobic was used, and a mixture of diesel and water in 1:2 ratios was poured through the device. The water easily penetrated through the mesh while diesel was blocked. Similarly, several other oils were also tested including gasoline, hexane, olive oil, mustard oil, and turpentine oil.

For the oil-removing mode, the mesh after hydrogen treatment was used. It has superhydrophobic/superoleophilic properties, making it possible to remove mixtures of chloroform and 1,2-dichloroethane with water. The oil quickly passed through the mesh while blocking the water. The contact angle measurements demonstrated the recoverability of the mesh for more than 10 cycles. Moreover, the efficiency of mesh was more than 99.9% for mustard oil, diesel, gasoline, olive oil, and turpentine oil.

## Future Perspectives

Although an understanding has been developed that superhydrophobic surfaces can be artificially created by combining nanoscale structures with hydrophobic functionality, most of these surfaces are susceptible to damage upon application of mechanical force. Therefore, efforts are still ongoing to make robust superhydrophobic surfaces. In most cases, the loss of durability is associated with destruction of nanoscale topography and chemical functionality under mechanical forces or harsh service conditions. Most of the durable superhydrophobic surfaces have been made by combining hydrophobic nanoparticles with adhesive; however, the resistance of such surfaces to organic solvents is still questionable. The response of a robust superhydrophobic surface in harsh environment such as pressure, wear, and shear has been discussed in a study; and it was argued that surface roughness plays a vital role in stability of Cassie–Baxter wetting state (Scarratt et al., 2017). A brief examination of the literature uncovers a deep concern regarding stability of superhydrophobic surfaces used for engineering applications and the methods to characterize them (Mortazavi and Khonsari, 2017). With increasing interest in the application of superhydrophobic surfaces for engineering materials, it has now become essential to devise standard methods to characterize the durability of superhydrophobic surface against different parameters such as mechanical or chemical attack.

In addition to the durability, special design considerations are required for the application of superhydrophobic materials. For example, most of the superhydrophobic materials in the literature are used for separation of oil/water mixtures where oil exists as a separate phase and can be separated by a simple filtration step. The separation becomes extremely difficult if the oil phase is uniformly distributed in the aqueous phase and stabilized by surfactants. In this case, the design of superhydrophobic materials becomes vital because typical absorbents are either inefficient or do not separate very stable emulsions. Despite some work present in the literature, this is still a developing field with a lot of unexplored facets. There is a dearth of characterization of emulsion separation by superhydrophobic materials. Most presented in the literature have used different recipes for emulsions, and in some cases, emulsions were not stable even up to 48 h.

A variety of materials have been reviewed for oil/water separation, and each of them has their strengths and weaknesses. For example, silica-based materials have been widely used in a variety of formulations, as their surface is rich with silanol groups and can be modified easily with other chemical moieties. Similarly, carbon soot provides an inexpensive route for large-scale production; however, the stability of soot-based materials is questionable. Other carbon-based materials such as CNTs and graphene composites offer dual-scale roughness due to their unique geometrical features. Iron-based oxides provide a unique advantage of reusability and fast separation due to their magnetic properties. It would be useful to explore techniques to make durable superhydrophobic composites, which can resist chemical/mechanical attack and offer some functional properties as well. Moreover, unconventional designs need to be considered for separation of surfactant stabilized emulsions.

## Author Contributions

All authors listed have made a substantial, direct and intellectual contribution to the work, and approved it for publication.

## Conflict of Interest

The authors declare that the research was conducted in the absence of any commercial or financial relationships that could be construed as a potential conflict of interest.
