# Supplementary material for: Surface Engineering of Ceramic Nanomaterials for Separation of Oil/Water Mixtures
Source: Front Chem. 2020 Nov 19;8:578. doi: 10.3389/fchem.2020.00578 (PMC7711160; doi:10.3389/fchem.2020.00578)
Supplement: Supplementary file 1 [file Data_Sheet_1.docx]

Surface engineering of ceramic nanomaterials for separation of oil/water mixtures

Usama Zulfiqar, Andrew Thomas, Allan Matthews,*

and David J. Lewis.*

^a^ Department of Materials and International Centre for Advanced Materials (ICAM, Manchester Hub), University of Manchester, Oxford Road, Manchester M13 9PL, UK

*Corresponding authors*

Professor Allan Matthews

[Allan.matthews@manchester.ac.uk](mailto:Allan.matthews@manchester.ac.uk)

Dr. David J. Lewis

[David.lewis-4@manchester.ac.uk](mailto:David.lewis-4@manchester.ac.uk)

Supplementary Information

Contents

[**Design parameters for the surface engineering of nanomaterials for physical separation of immiscible fluids** 3](#_Toc39027625)

[Surface wetting 3](#_Toc39027626)

[**Air/Water/Solid interface** 3](#_Toc39027627)

[**Oil/Water/Solid interface** 7](#_Toc39027628)

[**Progress in surface engineering of ceramic nanomaterials** 9](#_Toc39027629)

[Silica-based materials 9](#_Toc39027630)

[**Nanoparticles** 9](#_Toc39027631)

[Carbon 13](#_Toc39027632)

[**Graphene** 13](#_Toc39027633)

[**Carbon gels** 17](#_Toc39027634)

[**Titanium Oxide** 18](#_Toc39027635)

[**References** 21](#_Toc39027636)

# **Design parameters for the surface engineering of nanomaterials for physical separation of immiscible fluids**

##

## Surface wetting

### **Air/Water/Solid interface**

Wetting of a surface is an important phenomenon which can affect many functional properties of a material. It is important to first highlight the concepts of surface energy and surface tension before discussing the wetting of a surface. Surface energy (solid surfaces) and surface tension (fluid surfaces) originates from unsymmetrical bonding of the atoms present at the surface of solid or liquid. It can be ascribed as the energy required to generate a liquid or solid surface in vacuum or gas. It is measured in mN/m. This is actually the energy related to bonding with other atoms. When a liquid comes in contact with a surface there are several possibilities ranging from entirely spreading over the surface, to forming spherical droplets. The behavior of a liquid on a surface depends upon the physicochemical properties of the type of interface and is reflected by the angle formed by liquid droplet. For example, water will completely wet a surface of high surface energy e.g. glass (Simpson et al., 2015). On the other hand, water droplets tend to form a spherical shape on a low surface energy surfaces such as polytetrafluoroethylene (PTFE). Schematic (Fig. 2) shows the possible states of water droplet on a surface.


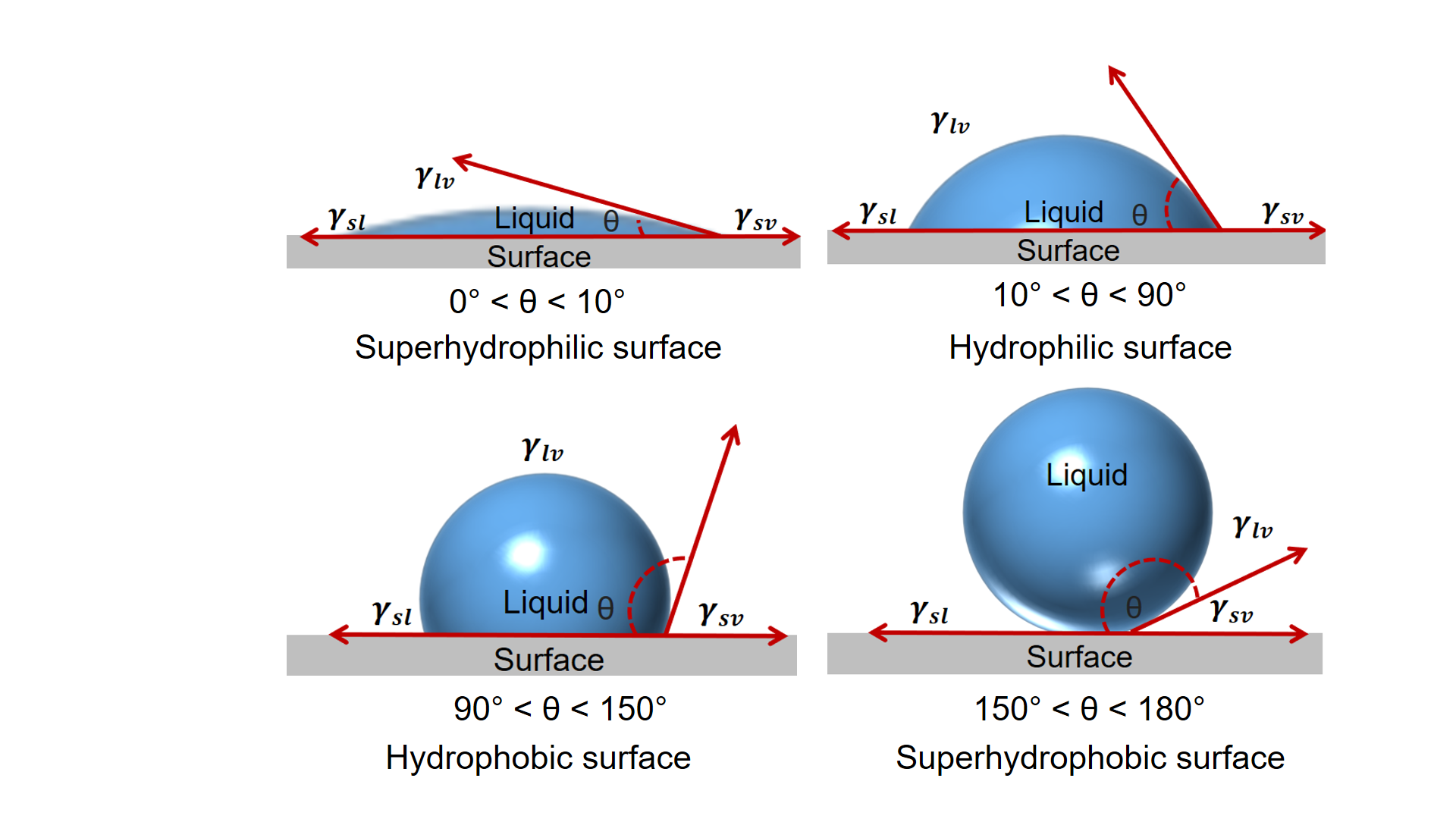


Figure S1: Schematic showing the four possible states of water droplet on a surface.

Traditionally, surface properties have been defined by the nature of their interaction with water. If a liquid droplet is suspended in air, its shape is determined by both gravity and the surface tension on liquid/vapor interface (Jeevahan et al., 2018). The liquid molecules at the interface are drawn inwards to maintain minimum surface area. The smallest surface area can be achieved by forming a sphere, however, gravitational pull acts to flatten the droplet. The effect of gravitational force can be ignored if the size of drop is less than the capillary length of water (2.7 mm). In this case, the shape of water drop will be determined solely by the surface tension of water (Shirtcliffe et al., 2010). Hence, if a small droplet (d < 2.7 mm) is placed on a flat surface there exists three different interfaces and the corresponding surface tensions. Young’s equation (Yong et al., 2017; Li et al., 2018)defines wettability under these conditions:

$\mathbf{cos}\boldsymbol{\theta=}\frac{\boldsymbol{\gamma}_{\boldsymbol{sv}}\boldsymbol{-}\boldsymbol{\gamma}_{\boldsymbol{sl}}}{\boldsymbol{\gamma}_{\boldsymbol{lv}}}$ (1)

Where:

*θº* = Contact angle of a water droplet on a flat surface;

*γ_sv_*= Interfacial tension of solid vapor phase;

*γ_sl_*= Interfacial tension of solid liquid phase;

*γ_lv_*= Interfacial tension of liquid vapor phase.

Young’s equation is derived by considering an ideally smooth surface, however, almost every surface has some inhomogeneity practically. The imperfections or defects contribute to the surface roughness and can greatly influence its wetting properties. Therefore, Wenzel proposed a modification to account for surface roughness in relation to contact angle:

$\boldsymbol{cos}\boldsymbol{\theta}_{\boldsymbol{w}}\boldsymbol{=r}\left( \frac{\boldsymbol{\gamma}_{\boldsymbol{sv}}\boldsymbol{-}\boldsymbol{\gamma}_{\boldsymbol{sl}}}{\boldsymbol{\gamma}_{\boldsymbol{lv}}} \right)$ (2)

Which by substitution into equation (1) becomes:

$\boldsymbol{cos}\boldsymbol{\theta}_{\boldsymbol{w}}\boldsymbol{=rcos\theta}$ (3)

And where:

*θ_w_*= Contact angle of the rough surface (Wenzel contact angle);

*θ*= Contact angle on a perfectly flat surface (Young’s contact angle).

*r* = Surface roughness

The Wenzel equation (Simpson et al., 2015) well explains the role of surface roughness in controlling the wettability of a surface. For a hydrophilic material, roughness will increase the wettability of a surface. Similarly, the roughness will further decrease the wettability of a hydrophobic surface. This equation not only includes rough surface but also explains the wettability of smooth surfaces. For example, if the roughness value (ratio actual surface versus projected surface r < 1 for a rough surface) is 1 (for a perfectly planar surface), the contact angle from Young’s equation is obtained.

The Cassie- Baxter model (Darmanin and Guittard, 2015; Simpson et al., 2015) proposes a heterogeneous structure for superhyrophobic surfaces. The water droplet rests on the asperities of a rough surface rather than entirely penetrating into the features, thus making a solid-liquid-air interface, and the following relationship applies: $\boldsymbol{cos\theta=}\boldsymbol{f}_{\boldsymbol{1}}\boldsymbol{cos}\boldsymbol{\theta}_{\boldsymbol{1}}\boldsymbol{+}\boldsymbol{f}_{\boldsymbol{2}}\boldsymbol{cos}\boldsymbol{\theta}_{\boldsymbol{2}}$ (4)

Where:

*θ* = Contact angle of a water droplet (Cassie-Baxter)

*f_1_* = Fraction of material 1 in contact with surface

*θ_1_* = Contact angle of a water droplet with surface

*f_2_* = Fraction of material 2 in contact with air

*θ_2_* = Contact angle of a water droplet with air

If the surface features have been made by one solid, then f1 represents the fraction of the solid which interacts with water and f_2_(1-f_1_) is the area fraction of air pockets in contact with the water droplet. Water contact angle with air is 180°, therefore the equation can be simplified to the following form:

$\boldsymbol{cos\theta=}\boldsymbol{f}_{\boldsymbol{s}}\left( \boldsymbol{1+cos}\boldsymbol{\theta}_{\boldsymbol{s}} \right)\boldsymbol{-1}$ (5)

Where f_s_ denotes the fractional area of solid with contact angle θ_s_ in contact with water droplet.

Therefore, increasing the roughness on a surface will increase its hydrophilic or hydrophobic properties depending upon the chemical composition of the surface. If a hydrophobic material is considered, the contact angle or water repellent properties of a surface will substantially increase if the surface features are capable of hosting air pockets in them. Thus, a hierarchically structured surface will give rise to a superhydrophobic surface owing to the surface roughness, air pockets and low surface energy chemistry. The schematic explains the behavior of a water droplet on a surface by all three models.


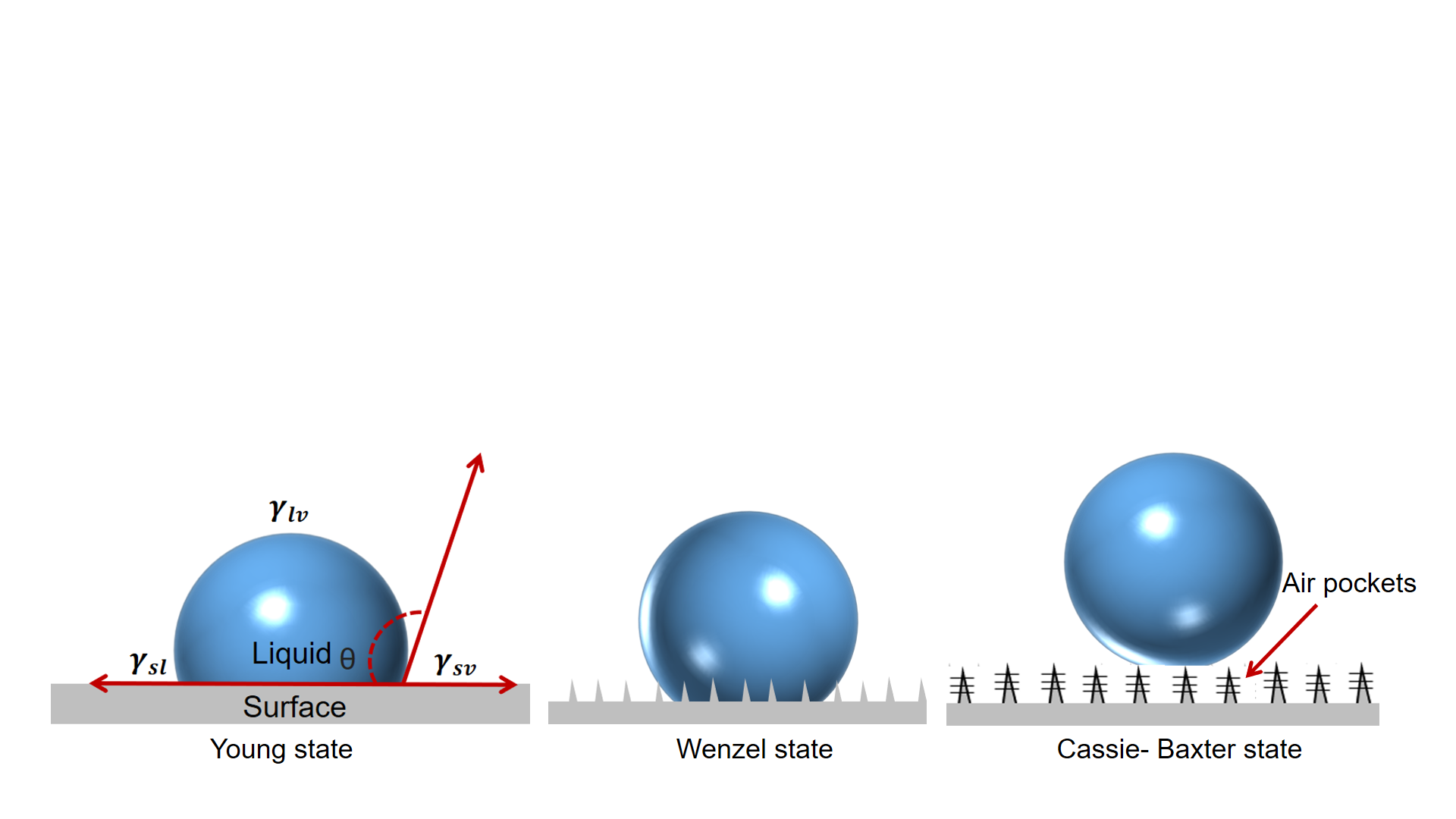


Figure S2: Schematic showing the behavior of water droplet in three different modals

### **Oil/Water/Solid interface**

For the application of superoleophobic surfaces in oil/water separation, it is important for them to possess an affinity towards water. Therefore, there has been recent trend in fabrication of underwater superoleophobic surface with superhydrophilic properties. These types of surfaces are generally engineered by infusing water into nanostructured surface. The water together with the rough texture blocks ingress of oil whilst letting water pass through. The corresponding oil contact angle formed in Oil/Water/Solid interface can be presented by the Young’s equation.

$\boldsymbol{cos}\theta_{ow}\boldsymbol{=}\frac{\boldsymbol{\gamma}_{\boldsymbol{sw}}\boldsymbol{-}\boldsymbol{\gamma}_{\boldsymbol{so}}}{\boldsymbol{\gamma}_{\boldsymbol{ow}}}$ **(6)**

*θow*= Contact angle of an oil droplet on a smooth surface in water

*γ_sw_*= Interfacial tension of solid water phase

*γ_so_*= Interfacial tension of solid oil phase

*γ_ow_*= Interfacial tension of oil water phase

Young’s equation (1) is valid for both oil and water droplets and can be written by replacing the values of *γ_sw_* and *γ_so_*. By replacing these values, we get the following equation:

$\boldsymbol{cos}\theta_{ow}\boldsymbol{=}\frac{\boldsymbol{\gamma}_{\boldsymbol{ov}\cos\text{θo}}\boldsymbol{-}\boldsymbol{\gamma}_{\boldsymbol{wv}\cos\boldsymbol{\theta w}}}{\boldsymbol{\gamma}_{\boldsymbol{ow}}}$ (7)

Hence a surface which is hydrophilic can become oleophobic in underwater conditions. In previous section, it was discussed in details that a heterogeneous interface (solid/air) is required to create superhydrophobic phenomenon. Similarly, the Wenzel and Cassie Baxter modals can be generalized for the solid water interface which means that an underwater rough surface with solid/water interface will exhibit superoleophobic properties (Li et al., 2018). The Wenzel model for underwater surfaces can be given as follows:

$\boldsymbol{cos}\boldsymbol{\theta}_{\boldsymbol{w}}\boldsymbol{=rcos}\boldsymbol{\theta}_{\boldsymbol{ow}}$ (8)

And Cassie Baxter modal

$\boldsymbol{cos}\boldsymbol{\theta}_{\boldsymbol{CB}}\boldsymbol{=}\boldsymbol{f}_{\boldsymbol{so}}\cos\boldsymbol{\theta}_{\boldsymbol{ow}}\boldsymbol{+}\boldsymbol{f}_{\boldsymbol{so}}\boldsymbol{-1}$ (9)

Similar to the superhydrophobic surface where air /solid interface help to increase the water contact angle, underwater superoleophobic surfaces exploit water/solid interface. The water layer sticks to the very rough structure of solid and provides superoleophobic character owing to its oil repellent nature.

# **Progress in surface engineering of ceramic nanomaterials**

## Silica-based materials

### **Nanoparticles**

In the reaction system, the tetraethyl orthosilicate (TEOS) poly(ethylene glycol) dimethacrylate (PEGDMA) and trimethylolpropanetris(3-mercaptopropionate) (TTMP) were used as reactants while ammonia/butylamine were used as two component catalyst. The silica nanoparticles were produced by the hydrolysis and condensation reaction of TEOS utilizing ammonia vapours as catalyst while the thiol-ene reaction took place in parallel by utilizing butylamine vapours as catalyst to form a cross linked hydrophilic polymer. The reaction was monitored by recording the ATR spectra at different time points and it was found that the reaction completes in 60 secs with the surface of silica nanoparticles covered with the hydrophilic thiolene polymer.

The surface composition was further confirmed by XPS. The carbon, oxygen, sulfur and silicon were found in 54.29 at. %, 33.46 at. %, 1.08 at. % and 11.17 at. %, respectively. After the completion of reaction, a rough morphology was observed by the SEM analysis of fabric. The roughness was induced by the deposition of silica particles which were generated from TEOS via a sol-gel reaction. AFM confirmed that the roughness was significantly increased after deposition of thiol-ene/silica hybrids as shown in 3D topographic representations. The root mean square (RMS) roughness of pristine fabric was 1.81 nm while the modified fabric showed a RMS value of 19.3 nm.

The fabric displayed underwater oil contact angles of more than 150° thus confirming its underwater superoleophobic properties. To separate the oil/water mixtures, the fabric was first prewetted by water then and then separation experiments were performed by using a mixture of hexane and water (7:3).

The water was separated from hexane which was blocked due to the superoleophobic property of the fabric. In addition, other mixtures of water with kerosene, soyabean, petroleum ether, chloroform, and 1,2-dichloroethane were separated by the same method with a separation efficiency more than 96%. The highest efficiency of 99.5% was achieved in case of 1,2-dichloroethane and chloroform. The water flux was also recorded, as shown in the. The variation in water flux value was ascribed to differences in solvent viscosity. The effect of temperature on separation efficiency and water flux was also studied and it was observed that efficiency increased with increasing temperature and an increasing trend was observed. It was suggested that the water molecules are more active at temperature which resulted in increase in water flux. It was further confirmed by calculating the water capture percentage (WCP) by the formula:

$$WCP \left( \% \right)=\frac{W_{b}-W_{a}}{W_{a}}\times100\%$$

where

*W_a_*= Initial mass of the fabric, *W_b_* = Mass of the fabric after dipping in water

The modified fabric shows higher WCP values demonstrating an improved ability to capture water as compared to pristine sample.

The recyclability was examined by separating the mixture of hexane with water for several cycles and it was found that the fabric maintained very high separation efficiency (98%) and water flux (63000 Lm^−2^h^−1^) even after 40 cycles of separation.

## Carbon

### **Graphene**

AFM revealed that GO sheets were in size range of 100–1500 nm with thickness of 1.2 nm . The XRD and XPS analysis of magnetic nanoparticles (MNP) indicated crystal structure and composition identical to γ-Fe2O3. The chemical structure of the nanomaterial produced was studied at each stage by FTIR. The peak corresponding to the vibration of silanol group was observed after modification of γ-Fe2O3 with TEOS and the peaks at 2963 cm-1 and 2925 cm-1 became stronger after modification with silane due to presence of –CH2. The chemical grafting of magnetic particle in M-GO was confirmed by the peaks associated with the deformation vibration of N-H or stretching vibration of C-N of the amide group (CO-NH-). The saturation magnetization of γ-Fe2O3 was decreased gradually after coating with silica and functionalization, however, the M-GO had good response towards external magnetic field which is essential for its application in oil/water separation.

Separation experiments were performed by using a crude oil in water emulsion which was stable up to 12 h. A small quantity of M-GO was introduced in the mixture which immediately changed its color indicating the breaking of the emulsion. Large oil droplets were quickly formed and came on the top of water to form a separate oil phase while the water phase displayed light yellow color which may have been caused by the presence of small oil droplets in the water. The sedimentation of small oil droplets was improved by using a magnet (Fig. S4b). Separation in a magnetic field made the water colorless within 5 min. Most of the M-GO was entrapped in the oil due to strong interaction, however, the M-GO were recovered by magnetic field and reactivated via washing with toluene. Micro morphologies of the emulsion before and after separation were obtained by polarized light microscope and it was found that the crude oil-in-water emulsion (5.0 wt%) contained the oil droplets (3–15 µm) uniformly distributed in the water phase. After demulsification, small amount of water droplets remained in the oil phase while the M-GO was present at the oil/water interface. In case of separated water, small floccules containing oil and M-GO were observed which could be eliminated with the application of a magnetic field. A demulsification efficiency of up to 99.98% and a reduction in residual oil concertation to 10 mg/L was achieved by increasing the dosage of M-GO to 0.25 wt%.

M-GO interacted with the asphaltene at the oil-water interface during separation, therefore, M-GO was washed with toluene before next separation cycle. Recyclability up to 6 cycle was measured with efficacy ca. 99.96% for first 6 cycles and <20 mg/L oil in separated water. The performance gradually decreases with most pronounced effect in the last 2 cycles where efficiency was decreased 93.5% and the oil concentration was increased to 550 mg/L. It is believed that natural surfactants such as asphaltenes were attached to the surface of M-GO and that washing with toluene was unable to remove them even after multiple cycles.

### **Carbon gels**

The PC fibers were activated in a sodium chlorite under acidic conditions followed by dispersion in ethanol. The SC-PC were dispersed in ethanol to retain the original network structure of the wet SC-PC gels during drying process. Wet SC-PC gel was obtained by filtering the suspension followed by oven drying at 60 °C. The obtained gels were carbonized at 1000 ° for 4 h under nitrogen atmosphere to obtain PSC aerogels. The formation mechanism of PSC aerogels from SC-PC aerogel was interpreted using TGA. It started with destruction of oxygen containing groups at 245−377 °C which correspond to the highest weight loss (∼76%). Later, the organic species converted to carbon and resulted in the formation of PSC aerogels. During the formation of PSC gels, about 91% weight loss of SC-PC aerogel was observed and the density was reduced to 4.3 mgcm^-3^ from 10 mgcm^-3^ before pyrolysis. The structural framework of PSC aerogel was entirely dependent upon the SC-PC aerogels as the SC treatment did not affect the morphology of PC fiber, however, the fibers experienced roughness and flattening after carbonization at 1000 °C in a N_2_ atmosphere.

XPS gave further information on transformation of raw PC fibers to PSC aerogels. The C/O atomic ratio was reduced to 3.62 from 7.67 after SC treatment which was caused by the oxidation of lignin and removal of impurities from the surface of the fibers. The carbonization resulted in the increased the C/O ratio to 12 which suggests that the most of the oxygen containing groups were removed from SC-OC aerogel. It was also observed form the high resolution C 1s spectra of the SC-PC and PSC aerogel that the C-O peak completely disappeared and a much stronger C-C peak was found. XRD further confirmed the formation of amorphous carbon after pyrolysis as the characteristic reflections of cellulose at 2θ=15.43º completely disappeared while the reflections 2θ= 22.42º was very weak.

### **Titanium Oxide**

SEM images reveal the morphology of the PLA membrane and it can be observed that micro-scale groves, microspores and stretched nanofibrils have created a hierarchical microstructure. A robust coating of TiO2@P(VP-VTES) was formed on the PLA membrane via spin coating. The TiO_2_ nanoparticles were seized by the stretched nanofibrils to form a coral tentacle -like structure. The high magnification images show that the TiO_2_ nanoparticles have completely covered the surface.

The surface composition was examined by EDX and XPS. EDX mapping showed the uniform distribution of TiO_2_ nanoparticles on the surface of membrane. The presence of Si and N confirmed the formation of copolymer P(VP-VTES). XPS spectra of coated membrane showed peaks of Ti 2p, N 1s and Si 2s/2p as compared to the spectra of pristine PLA membrane where only C 1s and O 1s were detected. This confirms that a high loading of P(VP-VTES) and TiO_2_ nanoparticles was anchored on the membrane surface.

Pristine PLA membrane had a water contact angle of 107° whilst the coated membrane showed superhydrophilic behavior. The difference in affinity towards water was ascribed to the high surface roughness (R_sa_ = 9.8 µm) of pristine membrane and the low surface roughness (5.6 µm) of the coated membrane.

The underwater superoleophobic contact angles were measured and it was found that the pristine membrane shows some affinity and adhesion towards droplets of 1,2-dichloroethane. The coated membrane demonstrated superoleophobic properties with an oil contact angle of 154±1° and very low adhesion force. It was suggested that a stable hydration layer was formed by the water entrapped in TiO_2_ coating and reduced the chances of contact between the membrane surface and oil. Therefore, this membrane could be a potential candidate for oil/water separation.

The coated membrane demonstrates low adhesion to oil and antifouling. It can be easily washed with water after repeated immersion in the heavy oil. It was used to separate many types of oil/water mixtures including toluene, paraffin, soybean and lubricating oil were separated with separation efficiencies of 1018 ± 16 L m^-2^ h^-1^, 1007 ± 16 L m^-2^ h^-1^, 989 ± 17 L m^-2^ h^-1^, and 963 ± 21 L m^-2^ h^-1^ , respectively with rejection higher than 99% . Moreover, the membrane maintained high flux, high separation efficiency and high rejection after ten separation cycles. In addition to the typical separation of oil/water mixtures, authors also characterized the membrane for anti-fouling properties against protein. FITC-labelled BSA was used for this purpose and almost no BSA adhesion was observed modified membrane in contrast to the pristine membrane. The pristine membrane showed the adsorption value of 17.8 ± 2.1 mg cm^-2^ while the adsorption value for modified mesh was very low 2.2 ± 0.6 mgcm^-2^.

# **References**

Darmanin, T., and Guittard, F. (2015). Superhydrophobic and superoleophobic properties in nature. *Mater. Today* 18, 273–285. doi:10.1016/J.MATTOD.2015.01.001.

Jeevahan, J., Chandrasekaran, M., Britto Joseph, G., Durairaj, R. B., and Mageshwaran, G. (2018). Superhydrophobic surfaces: a review on fundamentals, applications, and challenges. *J. Coatings Technol. Res.* 15, 231–250. doi:10.1007/s11998-017-0011-x.

Li, J.-J., Zhou, Y.-N., and Luo, Z.-H. (2018). Polymeric materials with switchable superwettability for controllable oil/water separation: A comprehensive review. *Prog. Polym. Sci.* 87, 1–33. doi:10.1016/J.PROGPOLYMSCI.2018.06.009.

Shirtcliffe, N. J., McHale, G., Atherton, S., and Newton, M. I. (2010). An introduction to superhydrophobicity. *Adv. Colloid Interface Sci.* 161, 124–138. doi:10.1016/J.CIS.2009.11.001.

Simpson, J. T., Hunter, S. R., and Aytug, T. (2015). Superhydrophobic materials and coatings: a review. *Reports Prog. Phys.* 78, 086501. doi:10.1088/0034-4885/78/8/086501.

Yong, J., Chen, F., Yang, Q., Huo, J., and Hou, X. (2017). Superoleophobic surfaces. *Chem. Soc. Rev.* 46, 4168–4217. doi:10.1039/C6CS00751A.
